# Anticancer Properties of Imidazolium Salt Derivatives: Current Advances and Therapeutic Perspectives

**DOI:** 10.3390/cancers18162556

**Published:** 2026-08-09

**Authors:** Diana Sawicka, Alicja Roztocka, Patryk Osiński, Jakub Nowak, Marta Pietruszyńska, Halina Car

**Affiliations:** 1 Department of Experimental Pharmacology, Medical University of Bialystok, Szpitalna Street 37, 15-295 Bialystok, Poland; halina.car@umb.edu.pl; 2 Student’s Pharmacological Club, Lazarski University, Świeradowska 43, 02-662 Warsaw, Poland; 18045ckp@lazarski.edu.pl (A.R.); 18044ckp@lazarski.edu.pl (P.O.); 46331@lazarski.edu.pl (J.N.); 3 Department of Clinical Pharmacology, Medical University of Bialystok, Waszyngtona 15A, 15-274 Bialystok, Poland; marta.pietruszynska@umb.edu.pl

**Keywords:** imidazolium salt derivatives, cancer, cytotoxicity, organic, non-organic substituents

## Abstract

Imidazolium salt derivatives (IMSDs) represent a structurally diverse class of compounds that has attracted increasing interest as a source of novel anticancer agents due to their tunable chemical architecture and broad spectrum of biological activities. This review summarizes current advances in the development of organic imidazolium derivatives and metal–N-heterocyclic carbene (NHC) complexes, with particular emphasis on their structure–activity relationships (SARs), mechanisms of action, and therapeutic potential. Numerous IMSDs exhibit significant antiproliferative activity against a wide range of cancer cell lines through multiple mechanisms, including DNA damage, reactive oxygen species generation, mitochondrial dysfunction, thioredoxin reductase inhibition, cell-cycle arrest, and apoptosis induction. Among the reported compounds, Au(I)-, Pt(II)-, and Ag(I)-NHC complexes consistently demonstrate the highest cytotoxic potency, whereas hybrid derivatives incorporating pharmacologically active moieties, such as lithocholic acid, have been investigated as potential approaches to improving selectivity and multitarget activity. Current SAR analyses indicate that metal coordination, bulky aromatic substituents, molecular hybridization, and balanced lipophilicity are key determinants of enhanced biological activity. Despite these encouraging findings, most available evidence is limited to in vitro studies, while comprehensive in vivo evaluation, pharmacokinetic characterization, and systematic toxicological assessment remain insufficient. Consequently, the clinical relevance of these compounds has yet to be established. Future research should focus on rational structural optimization, standardized preclinical evaluation, combination therapies, and advanced drug delivery strategies, including nanomedicine, to facilitate the translation of IMSDs into clinically useful anticancer agents.

## Simple Summary

Imidazolium salt derivatives are a diverse group of compounds that have attracted increasing interest as potential anticancer agents because their chemical structure can be readily modified to improve biological activity. Experimental studies have shown that many of these compounds inhibit cancer cell growth through multiple mechanisms, including DNA damage, oxidative stress, mitochondrial dysfunction, and apoptosis induction. Examples of compounds investigated in this context include metal–N-heterocyclic carbene complexes and hybrid derivatives containing biologically active molecules such as lithocholic acid. However, despite encouraging in vitro and limited in vivo findings, most imidazolium salt derivatives remain at the preclinical stage of development. Further studies are needed to evaluate their safety, pharmacokinetic properties, and therapeutic efficacy before their potential clinical application can be established. This review summarizes current advances, highlights structure–activity relationships, and discusses the main challenges that must be addressed to facilitate the future development of imidazolium-based anticancer therapies.

## 1. Introduction

**Figure 1:**
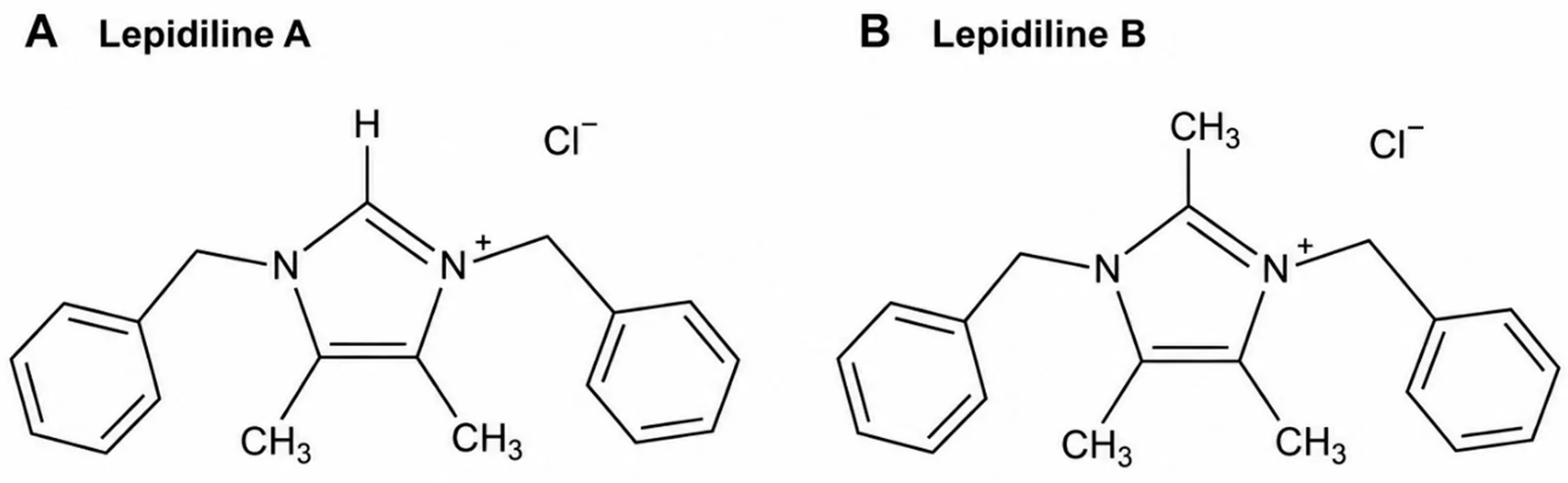
The structural formula of Lepidiline A (**A**) and Lepidiline B (**B**).

Imidazolium salt derivatives (IMSDs) are imidazole-based compounds containing an imidazolium cation formed via alkylation or related modifications of the imidazole ring. Such compounds have been identified in various natural products; for example, Lepidiline A and Lepidiline B (Figure 1A,B) were isolated from the roots of *Lepidium meyenii* [1,2,3,4].

It is well established that the imidazole ring, a fundamental structural element of numerous natural molecules, plays a crucial role in a wide range of biological structures and functions in the human body. It is present in the active sites of many enzymes, where it functions as both a proton donor and proton acceptor during biochemical reactions. The biological significance of imidazole-containing compounds arises from their broad spectrum of biological activities, highlighting the influence of the imidazole moiety on cellular and systemic functions, particularly in enzymatic catalysis, metal ion coordination, and pH buffering [5,6,7]. The functionality of the imidazole ring is well-documented, with its ability to bind to ligands and form hydrogen bonds being well-established. It is widely accepted that imidazolium salts (IMSs) are precursors of stable N-heterocyclic carbenes (NHCs) and bisimidazolidines, with a wide range of applications in organic synthesis [1,8]. Their widespread utility is primarily attributed to their exceptional structural tunability, as the NHC scaffold can be functionalized with a variety of substituents at different positions, allowing precise modulation of their physicochemical and biological properties [6]. IMSDs can be classified as ionic liquids when their melting point is below 100 °C and consist of discrete cations and anions. These compounds often exhibit low vapor pressure, high thermal and chemical stability, and tunable physicochemical properties, making them attractive substrates for a variety of industrial and biomedical applications [9]. The increasing application of IMSs as solvents in industrial chemical processes, separation and extraction technologies, and biological processes, has led to an increased emphasis on their biological properties, including toxicity, biodegradability, environmental persistence, and bioaccumulation [10]. One of the most attractive features of IMSs is the possibility of independently modifying both the cationic and anionic components, thereby generating compounds with diverse physical, chemical, and biological properties [11,12]. Such structural flexibility enables the incorporation of a wide range of organic and inorganic substituents, facilitating the development of compounds with enhanced biological activity, including anticancer properties. Furthermore, the presence of hydrophilic cationic moieties has been shown to promote interactions with essential biological targets biological components, such as cell membranes and DNA, thereby disrupting their structural integrity and biological functions [13].

Cancer remains one of the leading causes of morbidity and mortality worldwide, accounting for millions of deaths annually. Conventional treatment strategies, including surgery, chemotherapy and radiotherapy, are frequently insufficient for the effective management of many malignancies and are often associated with severe adverse effects, such as damage to healthy tissues, systemic toxicity and the development of multidrug resistance (MDR) [14,15]. Consequently, there is an urgent need to develop novel anticancer agents and more effective therapeutic strategies that improve treatment efficacy while reducing the toxicity associated with current chemotherapeutic regimens [16].

One of the principal objectives of medicinal chemistry is to improve the biological activity of lead compounds through the incorporation of bioactive pharmacophores. In recent decades molecular hybridization has emerged as an important strategy in drug discovery enabling the design of novel hybrid molecules with enhanced pharmacological properties [17]. In this context, the toxicity profile of IMSDs represents an important parameter that can be rationally optimized to support their development as potential therapeutic agents. Increasing attention has therefore been focused on the antitumor activities of IMSDs, which have demonstrated promising cytotoxic effects against various human cancer cell lines. Their anticancer activity has been associated with several mechanisms including those of G1 phase cell cycle arrest and activation of apoptosis pathways [18].

The present narrative review focuses on recent advances in the biological applications of IMSDs, with particular emphasis on their anticancer activity and their potential as promising candidates for the development of novel anticancer therapeutics.

### Literature Search Strategy

This work constitutes a narrative review of the literature. A search of the scientific literature was conducted in the PubMed, Scopus, and Web of Science databases to identify relevant studies concerning imidazolium salt derivatives.

The literature search was based on combinations of keywords related to imidazole chemistry and its derivatives, including terms such as “imidazole”, “imidazolium”, “metal–imidazole complexes”, and “organic and inorganic imidazole derivatives”, as well as terms related to biological and anticancer activity, such as “anticancer activity”, “cytotoxicity”, “in vitro”, and “in vivo”. The search was limited to publications from the last 20 years (2003–2026).

In addition to database searching, further relevant articles were identified through manual screening of the reference lists of selected publications. No formal inclusion or exclusion criteria were applied; instead, studies were selected based on their topical relevance and scientific contribution to understanding the anticancer potential of imidazolium salt derivatives. Duplicates were removed prior to analysis.

A total of 68 publications was included in the review. The selected literature comprised original research articles and review papers, which were used to provide a qualitative synthesis of current knowledge regarding the anticancer activity and proposed mechanisms of action of imidazolium salt derivatives.

## 2. IMSDS and Metal–NHC Substituents: Influence on Antitumor Activity

Imidazole salts represent fundamental precursors in the synthesis of carbenes (NHCs). These compounds are stable in their ionic form and, upon deprotonation, generate active NHC ligands capable of forming stable and catalytically active metal complexes. Initially, NHC complexes were developed and applied in homogeneous catalysis, where they exhibited excellent thermal and oxidative stability together with strong σ-donating properties [19]. However, over the past decade, their applications have expanded beyond catalysis, with increasing interest in their roles in biological systems, particularly in metal delivery and therapeutic applications. The ability of NHC ligands to form stable and well-defined complexes with transition metals has made them attractive candidates for biomedical use.

A wide variety of transition metals, including copper, silver, gold, platinum, palladium, and ruthenium, has been successfully coordinated with NHC ligands. These metal–NHC complexes often exhibit enhanced chemical stability and bioavailability, making them of interest for pharmaceutical development, especially in the search for novel anticancer and antimicrobial agents.

Recent studies have highlighted the potential anticancer activity of selected metal–NHC complexes [20,21]. The synthesis of NHC ligands typically begins with azolium salts, most notably imidazole derivatives which serve as precursors for generating free carbenes. These carbenes can then bind to various metal centers, forming complexes with diverse biological activities [3].

Among the most studied are silver–NHC complexes, which have shown not only potent antimicrobial effects but also significant cytotoxicity against various cancer cell lines. The scope of these activities is summarized in Table 1. Silver-containing NHC complexes have demonstrated inhibitory effects against multiple cancer types, including colon cancer [22,23,24,25,26], ovarian cancer [24,27,28,29], lung cancer [24,26,30,31], hepatocellular [32,33], melanoma [33], and breast cancer [22,23,24,25,26,31,34] and leukemia cells [35].

Of particular note is the complex p-bis((N-benzyl-benzimidazol-1-yl)methyl)benzene hexafluorophosphate silver(I), which exhibited high cytotoxicity against A2780 ovarian cancer cells, with a half maximal inhibitory concentration (IC_50_) value as low as 0.87 µM [24]. This level of potency indicates its potential relevance among silver–NHC complexes reported to date. The mechanisms of action of these complexes vary depending on the metal center and ligand structure, and are detailed in Table 1.

Recent investigations into the biological activity of transition metal–NHC (N-heterocyclic carbene) complexes have revealed promising antiproliferative properties across a range of metal centers. For example, iron–NHC complexes (Fe–NHCs) synthesized with the 1,3-bis(2,4,6-trimethylphenyl)imidazol-2-ylidene ligand were evaluated by Lenis-Rojas et al. for their cytotoxic effects against the ovarian cancer cell line A2780, the colon cancer cell line HCT 116, and normal human dermal fibroblasts [27]. These complexes demonstrated superior antiproliferative activity against HCT 116 cells when compared to reference drugs, including cisplatin. Notably, the IC_50_ values for these compounds were significantly lower for cancerous cells than for normal fibroblasts (IC_50_ = 23.6–42.04 µM), indicating selective toxicity and highlighting their therapeutic potential.

Copper-substituted imidazole-based NHC complexes have also been reported to exhibit potent cytotoxic effects, particularly against lung, breast, and prostate cancer cell lines [31]. Their activity underscores the versatility of NHC scaffolds in modulating the biological behavior of different transition metals.

Similarly, platinum-based NHC complexes have attracted considerable attention for their broad-spectrum anticancer activity. These compounds have been shown to inhibit cell proliferation of multiple cancer types, including colorectal cancer (HCT 116, HT-29), lung cancer (NCI-H460, A549), breast cancer (MDA-MB-231, MCF-7), ovarian cancer (A2780), hepatocellular carcinoma (HepG-2, Hepa1-6), and cisplatin-resistant ovarian cancer (A2780cis) [32,36]. A platinum(II) complex based on a C^N^N benzimidazole-derived NHC scaffold displayed high potency, with an IC_50_ of just 0.03 µM against A2780 ovarian cancer cells. While these complexes also showed some antiproliferative activity in normal cells such as lung fibroblasts (CCD-19Lu, IC_50_ = 3.26–4.94 µM) and hepatocytes (LO2, IC_50_ = 1.01–12.94 µM), their therapeutic window remains encouraging [32,36].

Ruthenium-based imidazole NHC complexes have also demonstrated cytotoxic potential, particularly against colorectal cancer, hepatocellular carcinoma cells [37], and lymphoma cell lines such as B-cell lymphoma 2 (Bcl-2) and B-cell lymphoma-extra-large (Bcl-xL) [38]. These findings suggest a role for Ru–NHCs in targeting hematological as well as solid tumors.

Gold–NHC complexes have emerged as some of the most potent candidates in this class of compounds. Their anticancer activity has been evaluated across several ovarian cancer cell lines (A2780, A2780/R, A2780/S, SKOV3, IGROV1), with bis(1-butyl-3-methyl-imidazole-2-ylidene)gold(I) hexafluorophosphate ([Au(NHC)_2_]PF_6_) exhibiting a low IC_50_ of 0.10 µM against A2780 cells [28,39,40]. Gold-based compounds have also demonstrated cytotoxic activity against a broad panel of lung cancer cell lines (A549, H522, H661, H1395, H1993, H157, H460, H1792, HCC-44, H1355), with IC_50_ values ranging from 0.95 to 91.00 µM [30]. Additional anticancer effects have been reported in melanoma [33], leukemia, and Burkitt lymphoma cell lines [35].

Importantly, some gold compounds have demonstrated selectivity for cancer cells. For example, Au(4,5-dichloro, N-methyl, N′-[2-hydroxyethylphenyl]imidazol-2-ylide), tested at a concentration of 5 µM, maintained 70% viability in normal human skin cells [33]. Imidazolyl gold(I) compounds evaluated on non-cancerous fibroblast cells (IMR90) showed relatively high IC_50_ values (63–87 µM), suggesting a promising therapeutic index [30].

**Figure 2:**
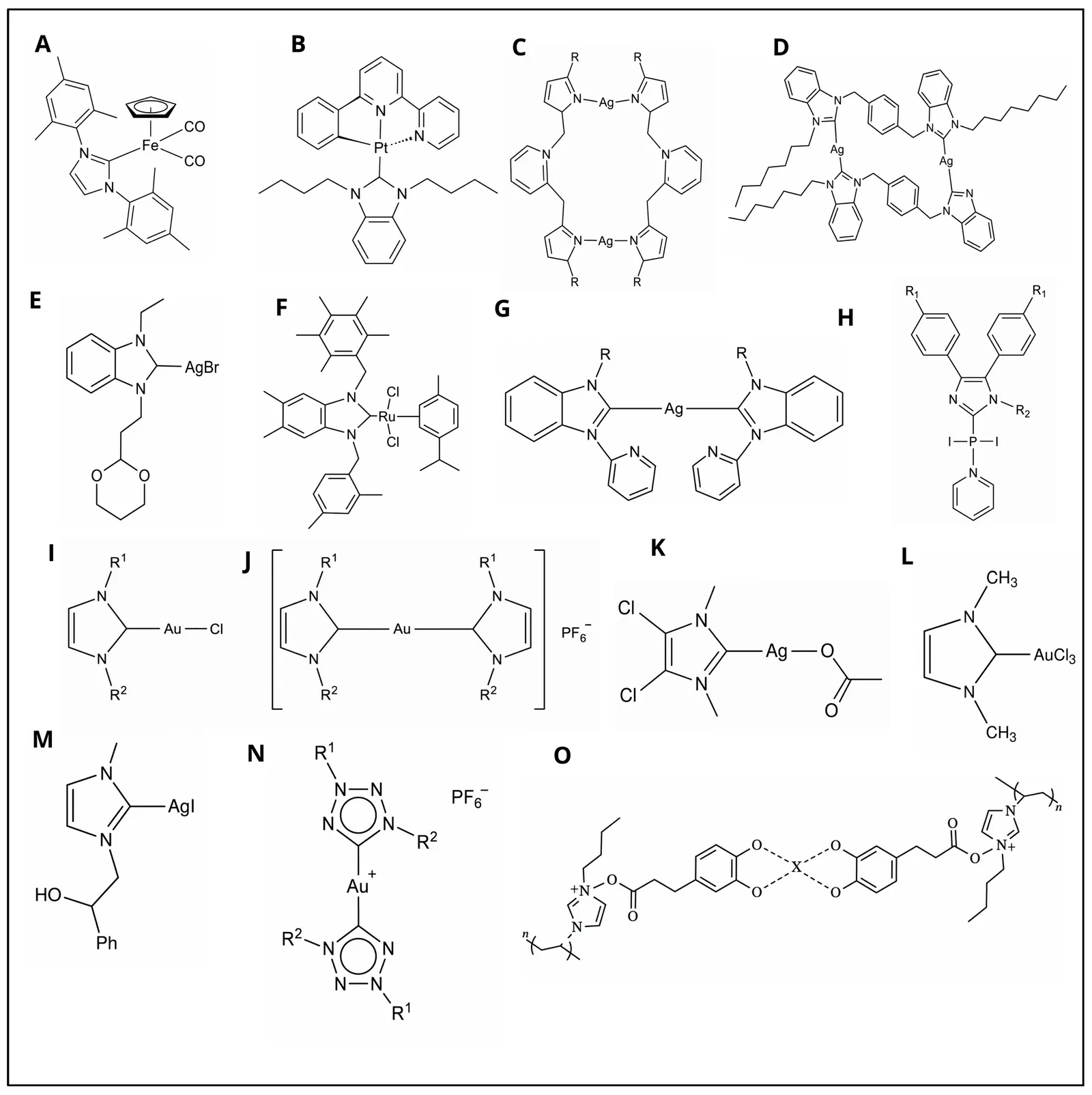
Structural formulas of the IMSDS compounds and metal–NHC complexes presented in Table 1. (**A**) Fe–NHC complexes, (**B**) Pt(II) complexes withselenophene, (**C**) Disilver Bis(hexafluorophosphate) (Ag(I)-NHC complexes with a meta-xylyl bridge) derivatives, (**D**) p-bis((N-heptane-benzimidazol-1-yl)methyl)benzene, hexa-flourophosphate silver, (**E**) Ag (I)-NHC complexes based on 1,3-dioxane ligand, (**F**) Ruthenium(II) N-Heterocyclic carbene complexes-benzimidazolium salts, (**G**) Silver N-heterocyclic biscarbene complexes, (**H**) Pt(II)-N-heterocyclic carbene complexes d rived from 4,5-diarylimidazole, (**I**) bis(1-butyl-3-methyl-imidazole-2-ylidene) gold(I), (**J**) chlorido (1-butyl-3-methyl-imidazole-2-ylidene) gold(I) [(NHC)AuCl], (**K**) Ag(I) N-heterocyclic carbene complexes obtained from 4,5-dichloro-1H-imidazole derivatives, (**L**) Imidazolyl Gold(I/III) compounds, (**M**) Ag(4,5-dichloro,N,N′-[2-hydroxy ethylphenyl]imidazol-2-ylide) derivatives, (**N**) Bis-(dimesityltetrazol-5-ylidene)gold(I) hexafluorophosphate derivative, (**O**) Halogen-free Metal-phenolic Imidazolium Poly(Ionic Liquids).

The specific anticancer mechanisms of these metal–NHC complexes, including their influence on apoptosis, DNA interaction, and mitochondrial pathways, are summarized in Table 1, whereas their corresponding chemical structures are shown in Figure 2A–O.

**Table 1:** IMSDs and metal–NHC complexes: structural influence on antitumor activity.

Type of Cancer	Experimental Model/Cell Lines	IMDs	IC_50_/Results/Mechanism/		IC_50_ of Reference Drug	Figure/Ref.
Colorectal cancer	HCT 116	Fe–NHC complexes	Cytotoxic effect IC50 = 0.87–3.26 µM Inhibition of cell proliferation (MTS assay)		Cisplatin IC_50_ = 15.6 µM	Figure 2A [27]
		Pt(II) complexes with selenophene, Pt(II) complex with thiophene and Pt(II) complex on a C^N^N scaffold, an NHC ligand derived from benzimidazole	Cytotoxic effect IC_50_ = 0.11– 0.15 µM Inhibition of cell proliferation (NBB Naphthol Blue-Black (NBB staining)		Cisplatin IC_50_ = 19.50 µM	Figure 2B [29]
		3,3′-[1,3-Phenylenebis(methylene)]bis(1-propyl-imidazolium) Disilver (I) Bis(hexafluorophosphate) (Ag(I)-NHC complexes with a meta-xylyl bridge) derivatives	Cytotoxic effect IC_50_ = 5.6–20.9 µM Inhibition of cell proliferation (MTT assay) The length of the alkyl chain influences the antiproliferative activity of the complexes, probably by facilitating the transport of Ag^+^ ions into cells, where they disrupt cellular metabolism and respiration.		5-fluorouracil IC_50_ = 5.9 µM	Figure 2C [23]
		Dinuclear Ag(I)-N-heterocyclic carbene complex (Ag(I)-NHC)	Cytotoxic effect IC_50_ = 20.9 μM Inhibition of cell proliferation (MTT assay) release of silver ions from the complex and binding of silver ions to DNA and proteins, interferes with cellular functions by interacting with enzymes and proteins			
		p-bis((N-heptane-benzimidazol-1-yl)methyl)benzene, hexa-flourophosphate silver (I), p-bis((N-benzy-benzimidazol-1-yl) methyl)benzene, hexa-flourophosphate silver (I)	Cytotoxic effect IC_50_ = 0.99–1.02 μM Inhibition of cell proliferation (MTT assay), inhibition tyrosine kinase (Bruton’s tyrosine kinase (BTK inhibition assay), ROS generation (Fluorescence microscopy assay)		ND	Figure 2D [24]
Colorectal cancer	HCT 116	Ag (I)-NHC complexes based on 1,3-dioxane ligand	Cytotoxic effect IC_50_ = 6.37–20.25 μM Inhibition of cell proliferation (MTT assay)		Cisplatin IC_50_ = 36.75 µM	Figure 2E [25]
		Ruthenium(II) N-Heterocyclic Carbene Complexes- benzimidazolium salts	Cytotoxic effect IC_50_ = 7.76–16.04 μM Inhibition of cell proliferation (MTT assay)		ND	Figure 2F [24]
	HT-29	Silver N-heterocyclic biscarbene complexes	Cytotoxic effect IC_50_ = 9.94–22.4 μM Inhibition of cell proliferation (MTT assay)		Cisplatin IC_50_ = 18.6 µM	Figure 2G [26]
		Silver N-heterocyclic monocarbene complexes	Cytotoxic effect IC_50_ = 10.4–11.4 μM Inhibition of cell proliferation (MTT assay)			
		Pt(II)-N-heterocyclic carbene complexes derived from 4,5-diarylimidazole	Cytotoxic effect IC_50_ = 1.73–19.71 μM Inhibition of cell proliferation (MTT assay)		Cisplatin IC_50_ = 5.71 µM	Figure 2H [32]
Hepatocellular carcinoma	HepG-2	Ruthenium(II) N-Heterocyclic Carbene Complexes- benzimidazolium salts	Cytotoxic effect IC_50_ = 11.75–26.82 μM Inhibition of cell proliferation (MTT assay)		ND	Figure 2F [24]
		Pt(II)-N-heterocyclic carbene complexes derived from 4,5-diarylimidazole	Cytotoxic effect IC_50_ = 0.33–14.6 μM	Inhibition of cell proliferation (MTT assay), immunogenic cell death inducer (ICD), which allows this imidazole to induce effective endoplasmic reticulum stress (ERS) with the generation of reactive oxygen species (ROS), which ultimately leads to the release of damage-associated molecular patterns (DAMPs) in cancer cells (Immunofluorescence assays)	Cisplatin IC_50_ = 2.68 µM	Figure 2H [32]
	Hepa1–6	Pt(II)-N-heterocyclic carbene complexes derived from 4,5-diarylimidazole	Cytotoxic effect IC_50_ = 0.36–6.56 μM		Cisplatin IC_50_ = 3.07 µM	
Ovarian carcinoma	A2780	Fe–NHC complexes	Cytotoxic effect IC_50_ = 1.34–2.91 µM Inhibition of cell proliferation (MTS assay)		Cisplatin IC_50_ = 1.9 µM	Figure 2A [27]
Ovarian carcinoma	A2780	Pt(II) complexes with selenophene, Pt(II) complex with thiophene and Pt(II) complex on a C^N^N scaffold, an NHC ligand derived from bezimidazole	Cytotoxic effect IC_50_ = 0.03–0.07 µM Inhibition of cell proliferation (NBB assay)		Cisplatin IC_50_ = 0.32 µM	Figure 2B [29]
		p-bis((N-heptane-benzimidazol-1-yl)methyl)benzene, hexa-flourophosphate silver derivatives	Cytotoxic effect IC_50_ = 0.87–0.96 μM Inhibition of cell proliferation (MTT assay), inhibition tyrosine kinase (Bruton’s tyrosine kinase (BTK inhibition assay). ROS generation (Fluorescence microscopy assay)		ND	Figure 2D [24]
		Pt(II)-N-heterocyclic carbene complexes derived from 4,5-diarylimidazole	Cytotoxic effect IC_50_ = 0.74–16.64 μM Inhibition of cell proliferation (MTT assay) immunogenic cell death inducer (ICD), which allows this imidazole to induce effective endoplasmic reticulum stress (ERS) with the generation of ROS, which ultimately leads to the release of damage-associated molecular patterns (DAMPs) in cancer cells (Immunofluorescence assays)		Cisplatin IC_50_ = 2.37 µM	Figure 2H [32]
		Bis(1-butyl-3-methyl-imidazole-2-ylidene) gold(I) ([Au(NHC)2]PF6)	Cytotoxic effect IC_50_ = 0.10 µM Inhibition of cell proliferation (MTT assay), induction of DNA fragmentation, cell cycle arrest in the G1 phase, activation of initiator caspases-8 and -9 and effector caspase-3 (Flow cytometry assays). Increased levels of glycolytic enzymes, metabolic shift toward glycolysis, and disruption of cellular respiration, inhibition of mitochondrial respiration		Cisplatin IC_50_ = 3.07 µM	Figure 2I [28,40]
		Chlorido (1-butyl-3-methyl-imidazole-2-ylidene) gold(I) [(NHC)AuCl]	Cytotoxic effect IC_50_ = 1.98 µM			Figure 2J [39]
Ovarian carcinoma	A2780	Bis(1-butyl-3-methyl-imidazole-2-ylidene) gold(I) ([Au(NHC)2]PF6) and chlorido (1-butyl-3-methyl-imidazole-2-ylidene) gold(I) [(NHC)AuCl] derivatives	Cytotoxic effect IC_50_ = 42.55–53.68 µM Inhibition of cell proliferation test SRB (Sulforhodamine B assay)		Cisplatin IC_50_ = 23.24 µM	Figure 2I [28,40]
	SKOV3	Bis(1-butyl-3-methyl-imidazole-2-ylidene) gold(I) ([Au(NHC)2]PF6) and chlorido (1-butyl-3-methyl-imidazole-2-ylidene) gold(I) [(NHC)AuCl]	Cytotoxic effect IC_50_ = 0.55–4.71 µM Inhibition of cell proliferation (MTT assay), Increased levels of glycolytic enzymes, metabolic shift toward glycolysis, and disruption of cellular respiration, inhibition of mitochondrial respiration TrxR		Cisplatin IC_50_ = 6.11 µM	Figure 2I [28]
	OVCAR-3	Ag(I) N-heterocyclic carbene complexes obtained from 4,5-dichloro-1H-imidazole derivatives	Cytotoxic effect IC_50_ = 20–35 µM Inhibition of cell proliferation (MTT assay)		Cisplatin IC_50_ = 12 µM	Figure 2K [34]
	IGROW1	Bis(1-butyl-3-methyl-imidazole-2-ylidene) gold(I) ([Au(NHC)2]PF6) and chlorido (1-butyl-3-methyl-imidazole-2-ylidene) gold(I) [(NHC)AuCl]	Cytotoxic effect IC_50_ = 0.39–3.92 µM Inhibition of TrxR Inhibition of cell proliferation (MTT assay), Increased levels of glycolytic enzymes, metabolic shift toward glycolysis, and disruption of cellular respiration, inhibition of mitochondrial respiration TrxR		Cisplatin IC_50_ = 1.17 µM	Figure 2I [28]
Lung cancer	A549	p-bis((N-heptane-benzimidazol-1-yl)methyl)benzene, hexa-flourophosphate silver (I), p-bis((N-benzy-benzimidazol-1-yl)methyl)benzene, hexa-flourophosphate silver (I)	Cytotoxic effect IC_50_ = 0.98–0.99 μM Inhibition of cell proliferation (MTT assay), inhibition tyrosine kinase (Bruton’s tyrosine kinase (BTK inhibition assay). ROS generation (Fluorescence microscopy assay)		ND	Figure 2D [24]
		Silver N-heterocyclic biscarbene complexes	Cytotoxic effect IC_50_ = 7.52–28.3 μM Inhibition of cell proliferation (MTT assay)		Cisplatin IC_50_ = 19.6 µM	Figure 2G [26]
		Silver N-heterocyclic monocarbene complexes	Cytotoxic effect IC_50_ = 7.06–10.1 μM Inhibition of cell proliferation (MTT assay)			
Lung cancer	A549	Pt(II)-N-heterocyclic carbene complexes derived from 4,5-diarylimidazole	Cytotoxic effect IC_50_ = 1.01–12.32 μM Inhibition of cell proliferation (MTT assay)		Cisplatin IC_50_ = 8.52 µM	Figure 2H [32]
		Imidazolyl Gold(I/III) Compounds	Cytotoxic effect IC_50_ = 9.1–91.0 μM	Inhibition of cell proliferation (MTT assay), Inhibition of mitochondrial respiration TrxR and Ligands DHFR (Enzymatic assays)	ND	Figure 2L [30]
	H522		Cytotoxic effect IC_50_ = 3.3–75.0 μM			
	H661		Cytotoxic effect IC_50_ = 0.95–47.0 μM			
	H1395		Cytotoxic effect IC_50_ = 5.9–62.0 μM			
	H1993		Cytotoxic effect IC_50_ = 1.8–70.0 μM			
	H157		Cytotoxic effect IC_50_ = 8.5–17.0 μM			
	H1792	Imidazolyl Gold(I/III) Compounds	Cytotoxic effect IC_50_ = 12.0–60.0 μM	Inhibition of cell proliferation (MTT assay), Inhibition of mitochondrial respiration TrxR and Ligands DHFR (Enzymatic assays)	ND	Figure 2L [30]
	HCC-44		Cytotoxic effect IC_50_ = 7.3–45.0 μM			
	H1355		Cytotoxic effect IC_50_ = 6.4–59.0 μM			
	H460		Cytotoxic effect IC_50_ = 6.6–68.0 μM			
		Pt(II) complexes with selenophene, Pt(II) complex with thiophene and Pt(II) complex on a C^N^N scaffold, an NHC ligand debrived from benzimidazole	Cytotoxic effect IC_50_ = 0.13–0.18 µM Inhibition of cell proliferation (MTT assay)		Cisplatin IC_50_ = 8.07 µM	Figure 2B [29]
Breast cancer	MDA-MB-231		Cytotoxic effect IC_50_ = 0.15–0.21 µM Inhibition of cell proliferation (NBB assay)		Cisplatin IC_50_ = 10.24 µM	
		Silver N-heterocyclic biscarbene complexes	Cytotoxic effect IC_50_ = 9.1–22.3 μM	Inhibition of cell proliferation (MTT assay)	Silver sulfadiazine IC_50_ = 11.2 µM	Figure 2G [26]
Breast cancer		Silver N-heterocyclic monocarbene complexes	Cytotoxic effect IC_50_ = 9.03–10.6 μM			
	MB157	Ag(I) N-heterocyclic carbene complexes obtained from 4,5-dichloro-1H-imidazole derivatives	Cytotoxic effect IC_50_ = 8–20 µM Inhibition of cell proliferation (MTT assay)		Cisplatin IC_50_ = 25 µM	Figure 2K [34]
	MCF-7	3,3′-[1,3-Phenylenebis(methylene)]bis(1-propyl-imidazolium) Disilver (I) Bis(hexafluorophosphate)(Ag(I)-NHC complexes with a meta-xylyl bridge) and 3,3′-[1,2-phenylenebis(methylene)]bis(1-propyl-imidazolium) Disilver (I) Bis(hexafluorophosphate) (Ag(I)-NHC complexes with an ortho-xylyl bridge)	Cytotoxic effect IC_50_ = 1.12–6.38 µM Inhibition of cell proliferation (MTT assay) The length of the alkyl chain influences the antiproliferative activity of the complexes, probably by facilitating the transport of Ag^+^ ions into cells, where they disrupt cellular metabolism and respiration		ND	Figure 2C [22]
		Pt(II)-N-heterocyclic carbene complexes derived from 4,5-diarylimidazole	Cytotoxic effect IC_50_ = 0.71–19.81 μM Inhibition of cell proliferation (MTT assay)		Cisplatin IC_50_ = 4.95 µM	Figure 2H [32]
		p-bis((N-heptane-benzimidazol-1-yl)methyl)benzene, hexa-flourophosphate silver (I), p-bis((N-benzy-benzimidazol-1-yl)methyl)benzene, hexa-flourophosphate silver (I)	Cytotoxic effect IC_50_ = 0.99–1.09 μM Inhibition of cell proliferation MTT assay, inhibition tyrosine kinase (Bruton’s tyrosine kinase (BTK inhibition assay). ROS generation (Fluorescence microscopy assay)		ND	Figure 2D [24]
		Ag (I)-NHC complexes based on 1,3-dioxane ligand	Cytotoxic effect IC_50_ = 3.72–8.14 μM Inhibition of cell proliferation (MTT assay)		Cisplatin IC_50_ = 32.17 µM	Figure 2E [25]
Breast cancer	MCF-7	Dinuclear Ag(I)-N-heterocyclic carbene complex (Ag(I)-NHC)	Cytotoxic effect IC_50_ = 2.4 μM Inhibition of cell proliferation (MTT assay), release of silver ions from the complex and binding of silver ions to DNA and proteins, interferes with cellular functions by interacting with enzymes and proteins (Synthesis DNA assay)		ND	Figure 2C [22]
Melanoma	MeWo	Ag(4,5-dichloro, N-methyl, N′-[2-hydroxy ethylphenyl]imidazol-2-ylide) derivatives	Cytotoxic effect IC_50_ = 5.84–24.63 Inhibition of cell proliferation (MTT assay), induction of apoptosis and DNA damage (Flow cytometry assay)		Cisplatin IC50 > 20 µM	Figure 2M [33]
Leukemia Cells	Nalm-6	Bis-(1,3-dimesityltetrazol-5-ylidene)gold(I) Hexafluorophosphate	Cytotoxic effect IC_50_ = 0.014 μM	Inhibition of cell proliferation (CASY Cell Counter assay), induction DNA fragmentation (Enzymatic assay), apoptosis and early necrosis, increase of ROS levels (Flow cytometry)	ND	Figure 2N [35]
		Bis-(1-*tert*-Butyl,3-*iso*-propyl-tetrazol-5-ylidene)gold(I) Hexafluorophosphate	Cytotoxic effect IC_50_ = 0.017μM			

IC_50_ (half maximal inhibitory concentration), ND not determined.

The cytotoxic activity of metal–NHC complexes is mediated through a multifaceted mechanism involving redox modulation, interference with nucleic acid function, and induction of programmed cell death pathways. For example, silver or gold NHC or complexes exhibit a strong propensity to interact with cellular DNA and nuclear proteins, leading to structural perturbations that compromise essential replication and transcription processes. These interactions ultimately result in cell-cycle arrest, predominantly within the G1 phase, thereby suppressing the proliferative capacity of malignant cells [33,35]. Concurrently, treatment with metal–NHC complexes has been observed to induce the generation of reactive oxygen species (ROS), culminating in elevated oxidative stress. The accumulation of ROS has been demonstrated to induce widespread macromolecular damage, particularly to nucleic acids, proteins, and membrane lipids, in addition to disrupting mitochondrial integrity [32,40]. A key molecular target implicated in this process is the selenoprotein thioredoxin reductase (TrxR), an enzyme crucial for maintaining the cellular redox equilibrium. Metal–NHC complexes have been shown to effectively inhibit TrxR activity, thereby exacerbating oxidative imbalance and amplifying ROS-mediated toxicity [28]. Furthermore, these complexes initiate the intrinsic (mitochondrial) apoptotic cascade, modulating members of the Bcl-2 protein family and promoting the activation of initiator and effector caspases (caspase-8, -9, and -3). This cascade consequently results in the controlled dismantling of the cellular architecture and the subsequent occurrence of apoptotic cell death. The induction of cell stress and death by metal–NHC complexes is associated with the release of damage-associated molecular patterns (DAMPs), which may act as immunogenic signals, thereby stimulating antitumor immune responses and contributing to the overall therapeutic efficacy [28,35,40]. It should also be noted that, in several of the cited studies, the investigated imidazolium complexes exhibited comparable or, in some cases, lower IC_50_ values than the reference drugs (Table 1). This suggests that these compounds may exert cytotoxic effects at relatively low concentrations, indicating in vitro antiproliferative activity. Overall, these findings highlight the potential of imidazolium complexes as interesting scaffolds for further investigation in anticancer research and future optimization.

Taken together, these findings lend support to a comprehensive model in which metal–NHC complexes exert potent antiproliferative effects through combined redox dysregulation, macromolecular targeting, and apoptotic signaling, thus underscoring their potential as promising chemotherapeutic candidates in the design of next-generation metal-based anticancer agents.

## 3. IMSDs with Hybrid IMSDs Incorporating Fused, Polycyclic or Natural-Product-Derived Pharmacophores: Influence on Antitumor Activity

**Figure 3:**
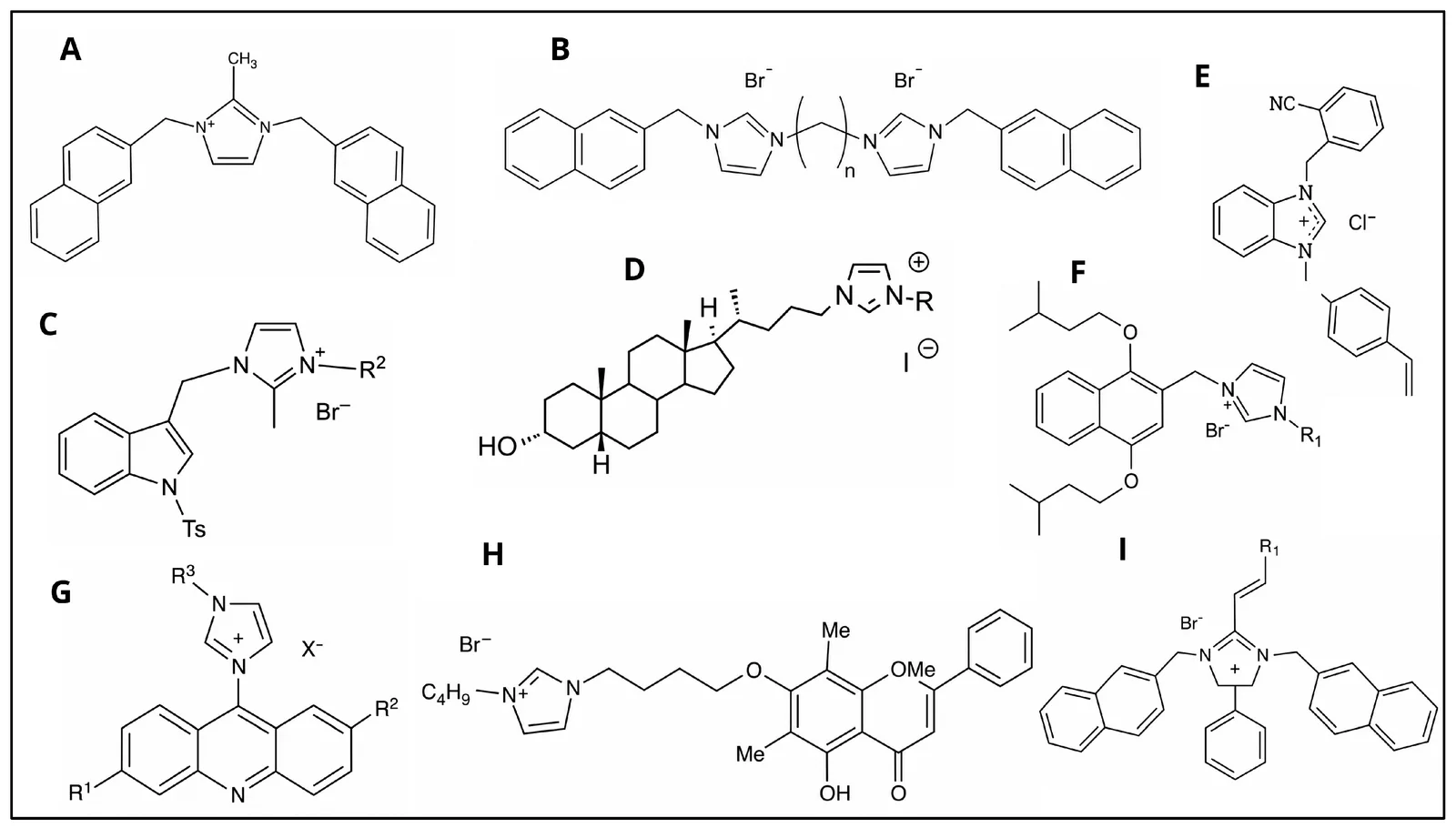
Chemical structures of hybrid imidazolium salts incorporating fused, polycyclic, or natural-product-derived pharmacophores described in Table 2. (**A**) Naphthylmethyl-substituted imidazolium salts, (**B**) N,N’-bis(naphthalen-2-ylmethyl)imidazolium with methyl/ethyl substitutions, (**C**) 1-((indol-3-yl)methyl)-1H-imidazolium salt, (**D**) Lithocholic acid (LCA)-based imidazolium salts, (**E**) Benzimidazolium salt (BS) (1-(2-cyanobenzyl)-3-(4 vinylbenzyl)-1H-benzo[d]imidazol-3-ium chloride), (**F**) 3-((1,4-Bis(isoamyloxy)naphthalen-2-yl)methyl)-1-phenyl-1H-imidazol-3-ium bromide, (**G**) Imidazolyl- acridine derivatives, (**H**) 4′-(4-bromobutoxy)-DMC conjugated with N-butylimidazole, (**I**) 2-(4-(Dimethylaminostyryl)-1,3-Bis(Naphthalen-2-Ylmethyl) benzimidazolium.

The design of hybrid imidazolium salts incorporating fused, polycyclic, or natural-product-derived pharmacophores has emerged as a promising strategy for the development of novel anticancer agents. The incorporation of biologically active molecular frameworks into the imidazolium core enables the combination of complementary pharmacological properties within a single molecule, often resulting in enhanced cytotoxicity, improved selectivity, and the ability to modulate multiple cellular targets. Depending on the nature of the incorporated pharmacophore, these hybrid compounds have been reported to induce apoptosis, interact with DNA, disrupt mitochondrial function, inhibit key enzymes, and interfere with signaling pathways involved in cancer progression. Table 2 summarizes representative hybrid imidazolium salts bearing fused, polycyclic, or natural-product-derived pharmacophores that have been evaluated for their anticancer activity, while the corresponding chemical structures are presented in Figure 3A–I. The compounds are grouped according to the type of cancer, together with the experimental models employed, cytotoxicity data (IC_50_, LD_50_, or GI_50_ values), and the mechanisms of action identified in the studies. This overview highlights the contribution of distinct pharmacophoric motifs to the biological performance of hybrid imidazolium salts and provides insight into the structure–activity relationships underlying their anticancer effects.

### 3.1. Naphthylmethyl-Substituted Imidazolium Salts

One of the most extensively investigated classes of imidazolium compounds comprises naphthylmethyl-substituted imidazolium salts. These salts are characterised by the presence of two bulky aromatic naphthalene rings attached to the imidazolium core. Among them, N,N′-bis(naphthalen-2-ylmethyl)imidazolium derivatives have demonstrated potent anticancer activity against several lung carcinoma cell lines, including A549, H460, H1975, and HCC827 [41,42]. The biological activity of these compounds is influenced by the nature of the alkyl substituents and the solvent environment. Derivatives bearing methyl, ethyl, propyl or butyl substituents at specific positions exhibited IC_50_ values below 5 μM, whereas the most active analogues demonstrated submicromolar potency (IC_50_ < 1 μM) in lung cancer models. A structure–activity relationship (SAR) was observed, with shorter alkyl chains generally associated with greater cytotoxic activity, suggesting that hydrophobicity and steric factors play a key role in regulating cellular uptake and mitochondrial accumulation.

Mechanistic studies revealed that these naphthylmethyl salts inhibit glycosyltransferase and galactosyltransferase enzymes, potentially disrupting glycoprotein biosynthesis, which is crucial for cancer cell proliferation. In addition, these compounds induce apoptosis and impair mitochondrial integrity, consistent with the lipophilic cationic nature of the imidazolium scaffold [41,42].

### 3.2. Indolyl-Substituted Imidazolium and Benzimidazolium Salts

Another recurring structural motif comprises indolyl-containing imidazolium and benzimidazolium cations, in which an indole moiety is linked to the heterocyclic core through either a methylene or an acyl bridge. The derivatives such as 1-((indol-3-yl)methyl)-1H-imidazolium and 1-((indol-3-yl)methyl)-3-(4-bromophenacyl)-5,6-dimethyl-1H-benzimidazolium salts, exhibited significant anticancer activities with IC_50_ = ranging from 3.23 to 5.51 μM against lung (A459)(breast MCF-7), liver (SMMC-7721) and colon (SW480)cancer cell lines [43].

Mechanistic studies demonstrated that these compounds inhibit cell cycle progression by inducing S-phase arrest, activate caspase-dependent apoptosis, and disrupt mitochondrial membrane potential. Furthermore, structural modifications involving the incorporation of bromophenacyl or naphthylacyl substituents further enhanced their antiproliferative activity, suggesting that electron-withdrawing groups and extended aromatic conjugation improve DNA-binding affinity [43].

In addition, an indolyl-benzimidazolium derivative bearing a 4-methoxyphenacyl substituent exhibited remarkable cytotoxic activity against HL-60 leukemia cells, with an IC_50_ value of 1.89 μM, indicating a potent pro-apoptotic effect [43].

### 3.3. Acridinyl-Imidazolium Derivatives

Acridine-substituted imidazolium derivatives represent a class of compounds investigated for their potential anticancer activity, combining a planar heteroaromatic chromophore with the cationic imidazolium unit. Compounds such as 1-(2-methoxyacridin-9-yl)-3-methylimidazolium chloride and 9-(1-imidazolyl)acridine have demonstrated broad-spectrum antiproliferative activity against ovarian (CAOV-3), prostate (PC-3), and breast (MCF-7) cancer cell lines, with IC_50_ values ranging from 2.5 to 9 μg/mL [44]. Mechanistic studies suggest that their anticancer activity is primarily associated with DNA intercalation as confirmed by fluorescence-based DNA-binding assays, together with cytotoxicity and redox-modulating studies. Intriguingly, the incorporation of a methoxy substituent into the acridine moiety reduced the antioxidant capacity of these compounds, suggesting an inverse relationship between radical-scavenging activity and cytotoxic potency. Furthermore, derivatives such as 1-(6-chloro-2-methoxyacridin-9-yl)-3-benzylimidazolium chloride exhibited enhanced DNA-binding affinity and a greater ability to induce apoptosis, with IC_50_ = 5 μg/mL.

### 3.4. Lithocholic Acid (LCA)-Based Imidazolium Salts

The incorporation of a steroidal backbone from lithocholic acid (LCA) into imidazolium salts has led to the development of a structurally unique family of amphiphilic cations [45]. A series of imidazolium salts differing in the length of the alkyl chain (C1–C16) was synthesized and evaluated in vitro against colorectal adenocarcinoma cell lines (DLD-1 and HT-29). These compounds reduced cellular metabolism and viability, with IC_50_ values ranging from 78.9 to 88.4 µg/mL, and induced apoptosis through the activation of Bcl proteins and the tumor necrosis factor-α (TNF-α) signaling pathway. The anticancer activity of these compounds was also investigated against breast cancer cell lines (MCF-7 and MDA-MB-231). Among the tested derivatives, the imidazolium salt S8, containing an eight-carbon alkyl chain, exhibited the highest cytotoxic activity, with IC_50_ values of 13.19 µg/mL for MCF-7 cells and 21.06 µg/mL for MDA-MB-231 cells, outperforming the reference chemotherapeutic agent doxorubicin. Mechanistic studies demonstrated that S8 inhibits DNA synthesis and induces both apoptosis and necrosis in breast cancer cells. Furthermore, combination treatment with S8 and doxorubicin produced a strong synergistic effect in MCF-7 cells, as indicated by a fractional inhibitory concentration index (FICI) of 0.68 and a markedly reduced IC_50_ value of 0.68 µg/mL. In contrast, the same combination exhibited an antagonistic interaction in MDA-MB-231 cells, with a FICI of 1.87 and an IC_50_ value of 63.69 µg/mL [46]. The biological activity of these compounds appears to result from the combined contribution of the hydrophobic lithocholic acid scaffold and the cationic imidazolium head group. Together, these structural features enhance interactions with cellular membranes and promote the activation of apoptotic signaling pathways, thereby contributing to their anticancer effects.

### 3.5. Benzimidazolium Derivatives with Extended Conjugation

A number of benzimidazolium derivatives, incorporating aromatic vinyl or naphthyl substituents, has been observed to demonstrate potent antiproliferative activity. This effect has been attributed to the presence of extended π-conjugated systems, which facilitate DNA intercalation.

For example, 1-(2-cyanobenzyl)-3-(4-vinylbenzyl)-1H-benzimidazol-3-ium chloride exhibited IC_50_ values ranging from 20 to 40 μM and induced cell cycle arrest in the S and G2/M phases through a caspase-dependent apoptotic pathway [47]. Furthermore, styryl-substituted bis(naphthalen-2-ylmethyl)benzimidazolium bromides exhibited remarkable anticancer activity in the NCI-60 human cancer cell panel, with GI_50_ values below 0.6 μM across multiple cancer cell lines [48].

The introduction of methoxy, methyl, or ethoxy substituents on the styryl moiety was found to fine-tune the electronic properties of the conjugated system, revealing a clear SAR. These findings suggest that modulation of the electronic characteristics of the aromatic substituents enhances anticancer activity by strengthening π–π stacking and charge-transfer interactions with biological targets. Mechanistic studies demonstrated that these highly conjugated cationic compounds act as DNA intercalators and disrupt mitochondrial function, highlighting the synergistic contribution of extensive π-electron delocalization and the positively charged benzimidazolium core to their anticancer activity [48].

### 3.6. Alkoxy-Substituted Imidazolium Halides and Dimethylcurcumin Conjugates

Imidazolium conjugated with 4′-(4-bromobutoxy) dimethylcurcumin (DMC), the resulting hybrid molecules enhanced anticancer activity against breast cancer cells, exhibiting IC_50_ values ranging from 4.9 to 7.4 μM and improved selectivity indices (SI = 1.4–2.1). Mechanistic studies demonstrated that these conjugates induce DNA damage, promote mitochondrial depolarization, and upregulate the expression of the pro-apoptotic genes Bax and p53 while downregulating the anti-apoptotic gene Bcl-2, thereby confirming the involvement of the intrinsic mitochondrial apoptotic pathway [49].

**Table 2:** Hybrid IMSDs incorporating fused, polycyclic or natural-product-derived pharmacophores: structural influence on antitumor activity.

Type of Cancer	Experimental Model/ Cell Lines	IMSDs	IC_50_/LD_50_/GI_50_/Results/Mechanism		IC_50_ of Reference Drug	Figure/Ref.
Lung cancer	H460	Naphthylmethyl-substituted imidazolium salts	Cytotoxic effect IC_50_ < 1 μM	Inhibition of cell proliferation (MTT assay) Inhibit human galactosyltransferase and glycosyltransferase enzymes (Enzymatic assays)	Cisplatin IC_50_ = 6 μM	Figure 3A [42]
		(Me w/DMSO)- N,N’-bis(naphthalen-2-ylmethyl)imidazolium with methyl/ethyl substitutions	Cytotoxic effect IC_50_ = 2.53–2.70 μM	Inhibition of cell proliferation (MTT assay). The lipophilicity of the compounds correlated with their cytotoxic activity; longer alkyl chains generally reduced cell growth, possibly through mitochondrial dysfunction (Microscopy analysis)	Cisplatin IC_50_ = 2.92 μM	Figure 3B [41]
	H1975	Naphthylmethyl-substituted imidazolium salts	Cytotoxic effect IC_50_ < 1 μM	Inhibition of cell proliferation (MTT assay) Inhibit human galactosyltransferase and glycosyltransferase enzymes (Enzymatic assays)	Cisplatin IC_50_ = 3 μM	Figure 3A [42]
		N,N’-bis(naphthalen-2-ylmethyl)imidazolium with methyl/butyl substitutions	Cytotoxic effect IC_50_ = 0.66–1.72 μM	Inhibition of cell proliferation (MTT assay) Induction of cell’s apoptosis (Flow cytometry)	Cisplatin IC_50_ = 9.77 μM	Figure 3B [41]
	HCC827		Cytotoxic effect IC_50_ = 1.15–2.64 μM		Cisplatin IC_50_ = 4.76 μM	
Lung cancer	HCC827	Naphthylmethyl-substituted imidazolium salts	Cytotoxic effect IC_50_ = 4 μM	Inhibition of cell proliferation (MTT assay) Inhibit human galactosyltransferase and glycosyltransferase enzymes (Enzymatic assays)	Cisplatin IC_50_ = 9 μM	Figure 3A [42]
	A549		Cytotoxic effect IC_50_ < 1 μM		Cisplatin IC_50_ = 5 μM	
		N,N’-bis(naphthalen-2-ylmethyl)imidazolium with methyl/butyl substitutions	Cytotoxic effect IC_50_ = 2.13–2.63 μM	Inhibition of cell proliferation (MTT assay), induction of cell ‘s apoptosis (Flow cytometry)	Cisplatin IC_50_ = 5.63 μM	Figure 3B [41]
		1-((indol-3-yl)methyl)-1H-imidazolium salt	Cytotoxic effect IC_50_ = 3.51 μM	Inhibition of cell proliferation (MTT assay). Block the S-phase of the cell cycle and induce cell apoptosis (Flow cytometry)	Cisplatin IC_50_ = 6.01 μM	Figure 3C [43]
Colorectal adenocarcinoma	DLD-1	Lithocholic acid (LCA)-based imidazolium salts with differing in the number of carbon atoms C1–C16 in the alkyl chain	Cytotoxic effect IC_50_ = 78.9 μg/mL-for S6 (with C6 in alkyl chain)	Inhibition of cell proliferation (MTT assay) Induction of apoptosis (activation of Bcl proteins and TNF-α), reduction in the cellular metabolism and viability (Enzymatic assays)	ND	Figure 3D [45]
		Benzimidazolium salt (BS) (1-(2-cyanobenzyl)-3-(4 vinylbenzyl)-1H-benzo[d]imidazol-3-ium chloride)	Cytotoxic effect IC_50_ = 40 μM Inhibition of cell proliferation (MTT assay), Induction of cells apoptosis (Flow cytometry)			Figure 3E [47]
Colorectal adenocarcinoma	HT29	Lithocholic acid (LCA)-based imidazolium salts with differing in the number of carbon atoms C1–C16 in the alkyl chain	Cytotoxic effect IC_50_ = 88.4 μg/mL for S4 (with C4 in alkyl chain) Inhibition of cell proliferation (MTT assay)		ND	Figure 3D [45]
		3-((1,4-Bis(isoamyloxy)naphthalen-2-yl)methyl)-1-phenyl-1H-imidazol-3-ium bromide	Cytotoxic effect LD_50_ = 6.09 μM Inhibition of cell proliferation (WST assay Water-soluble tetrazolium salt assay)			Figure 3F [50]
		Benzimidazolium salt (BS) (1-(2-cyanobenzyl)-3-(4vinylbenzyl)-1H-benzo[d]imidazol-3-ium chloride)	Cytotoxic effect IC_50_ = 20 μM Inhibition of cell proliferation (MTT assay). induction of cells apoptosis, caspase-dependent mechanism, the greatest impact on the cells was observed in the G2-M phase (Flow cytometry)		ND	Figure 3E [47]
	SW480	1-((indol-3-yl)methyl)-3-(4-bromophenacyl)-5,6-dimethyl-1H-benzimidazolium salt	Cytotoxic effect IC_50_ = 5.51 μM Inhibition of cell proliferation (MTT assay) Block the S-phase of the cell cycle and induce cell apoptosis (Flow cytometry)		Cisplatin IC_50_ = 16.3 μM	Figure 3C [43]
Ovarian cancer	CAOV-3	(1-(2-Methoxyacridin-9-yl)-3-methylimidazolium chloride)	Cytotoxic effect IC_50_ = 2.5 μg/mL	Inhibition of cell proliferation (MTT assay), antioxidant activity, intercalation DNA (DPHH assay)	Tamoxifen IC_50_ = 2 μg/mL Paclitaxel IC_50_ = 4.8 μg/mL	Figure 3G [44]
Prostate cancer	PC-3	9-(1-Imidazolyl) acridine	Cytotoxic effect IC_50_ = 6 μg/mL		Tamoxifen IC_50_ = 2 μg/mL Paclitaxel IC_50_ = 5 μg/mL	
Breast cancer	MCF-7	1-(6-Chloro-2-methoxyacridin-9-yl)-3-benzylimidazolium chloride	Cytotoxic effect IC_50_ = 5 μg/mL		Tamoxifen IC_50_ = 11 μg/mL Paclitaxel IC_50_ = 6 μg/mL	
		1-(Acridin-9-yl)-3-hexylimidazolium chloride	Cytotoxic effect IC_50_ = 9 μg/mL			
		1-((indol-3-yl)methyl)-3-(4-bromophenacyl/naphthylacyl)-5,6-dimethyl-1H-benzimidazolium salt	Cytotoxic effect IC_50_ = 3.16–3.70 μM Inhibition of cell proliferation (MTT assay) Block the S-phase of the cell cycle and induce cell apoptosis (Flow cytometry)		Cisplatin IC_50_ = 15.38 μM	Figure 3C [43]
Breast cancer	MCF-7	Lithocholic acid (LCA)-based imidazolium salts with differing in the number of carbon atoms C1–C16 in the alkyl chain	Cytotoxic effect IC_50_ = 13.19 μg/mL for S8(with C8 in alkyl chain)	Inhibition of cell proliferation (MTT assay) the salts tested reduced the viability and DNA synthesis. Strong induction of apoptosis and necrosis (Enzymatic assays)	Doxorubicin IC_50_ = 26.18 μg/mL Synergistic effect for S8 and Dox IC_50_ = 11.98 μg/mL	Figure 3D [46]
	MDA-MB-231	4′-(4-bromobutoxy)-DMC conjugated with N-butylimidazole	Cytotoxic effect IC_50_ = 4.95 μM	Inhibition of cell proliferation (MTT assay), DNA damage, leading to apoptosis, the loss of mitochondrial membrane permeability leads to an increase in the expression of Bax/Bcl-2 and p53 genes (Enzymatic assays)	Doxorubicin IC_50_ = 0.38 μM	Figure 3H [49]
			Cytotoxic effect IC_50_ = 7.40 µM		Doxorubicin IC_50_ = 1.07 μM	
		Lithocholic acid (LCA)-based imidazolium salts with differing in the number of carbon atoms C1–C16 in the alkyl chain	Cytotoxic effect IC_50_ = 21.06 μg/mL for S8 (with C8 in alkyl chain)	Inhibition of cell proliferation (MTT assay) the salts tested reduced the viability and DNA synthesis. Strong induction of apoptosis and necrosis (Enzymatic assays)	Doxorubicin IC_50_ = 90.0 μg/mL Antagonistic effect and Dox for salts IC_50_ = 63.69 μg/mL	Figure 3D [46]
Liver carcinoma	HepG2	1-Benzyl-3-(2-(1,4-bis(isoamyloxy)naphthalen-2-yl)ethyl)-1H-imidazol-3-ium bromide	Cytotoxic effect LD_50_ = 6.20 μM	Inhibition of cell proliferation (WST assay)	ND	Figure 3F [50]
	SMMC-7721	1-((indol-3-yl)methyl)-1H-imidazolium salts	Cytotoxic effect IC_50_ = 3.60 μM	Inhibition of cell proliferation (MTT assay) Block the S-phase of the cell cycle and induce cell apoptosis (Flow cytometry)	Cisplatin IC_50_ = 6.76 μM	Figure 3C [43]
Leukaemia	HL-60		Cytotoxic effect IC_50_ = 1.89 μM		Cisplatin IC_50_ = 1.05 μM	
Human Tumor Cell Line Screen	NCI-60	2-(4-(Dimethylaminostyryl)-1,3-Bis(Naphthalen-2-Ylmethyl) benzimidazolium bromide derivatives	Cytotoxic effect GI_50_ = 0.29–0.59 µM Inhibition of cell proliferation (Sulforhodamine B, SRB assay)		ND	Figure 3I [48]

IC_50_ (half maximal inhibitory concentration), GI_50_ (50% growth inhibition vs. control), LD_50_ (median lethal dose), ND not determined.

Hybrid imidazolium salts incorporating fused polycyclic systems or natural product-derived pharmacophores exert their anticancer activity through multiple interconnected molecular mechanisms while often exhibiting selective toxicity toward cancer cells, thereby minimizing damage to healthy tissues. One of the principal mechanisms involves mitochondrial dysfunction, which activates intrinsic apoptotic signaling cascades, including caspases, Bcl family proteins, and TNF-related pathways, leading to programmed cell death [45]. Furthermore, increased generation of ROS promoted oxidative stress, further contributing to apoptosis [44]. Several hybrid IMSDs have also demonstrated to induce cell cycle arrest at the S and G2/M phases, thereby impairing DNA replication and mitotic division [41,43,47]. Their interaction with DNA including intercalation by extended aromatic systems, together with activation of the p53 signaling pathway, enhances the cellular DNA damage response and promotes apoptosis, reinforcing their antiproliferative activity [42]. The diverse mechanisms of action of these hybrid compounds act synergistically to disrupt essential cellular processes, suppress cancer cell proliferation, and induce apoptotic cell death. In several of the studies reviewed, the investigated derivatives exhibited IC_50_ values comparable to or lower than those of conventional chemotherapeutic agents, including cisplatin and doxorubicin, indicating potent antiproliferative activity under the tested in vitro conditions. Moreover, in several of the referenced studies, the investigated imidazolium complexes showed IC_50_ values comparable to or, in some cases, lower than those reported for standard chemotherapeutic agents such as cisplatin and doxorubicin. These findings suggest that, under the tested in vitro conditions, certain compounds may exhibit cytotoxic activity at relatively low concentrations, indicating promising antiproliferative properties against malignant cells. Collectively, these findings demonstrate that the incorporation of fused aromatic systems and natural product-derived pharmacophores into the imidazolium scaffold represents an effective strategy for enhancing anticancer activity.

## 4. Structurally Modified IMSDs Bearing Aromatic and Alkyl Substituents: Influence on Antitumor Activity

**Figure 4:**
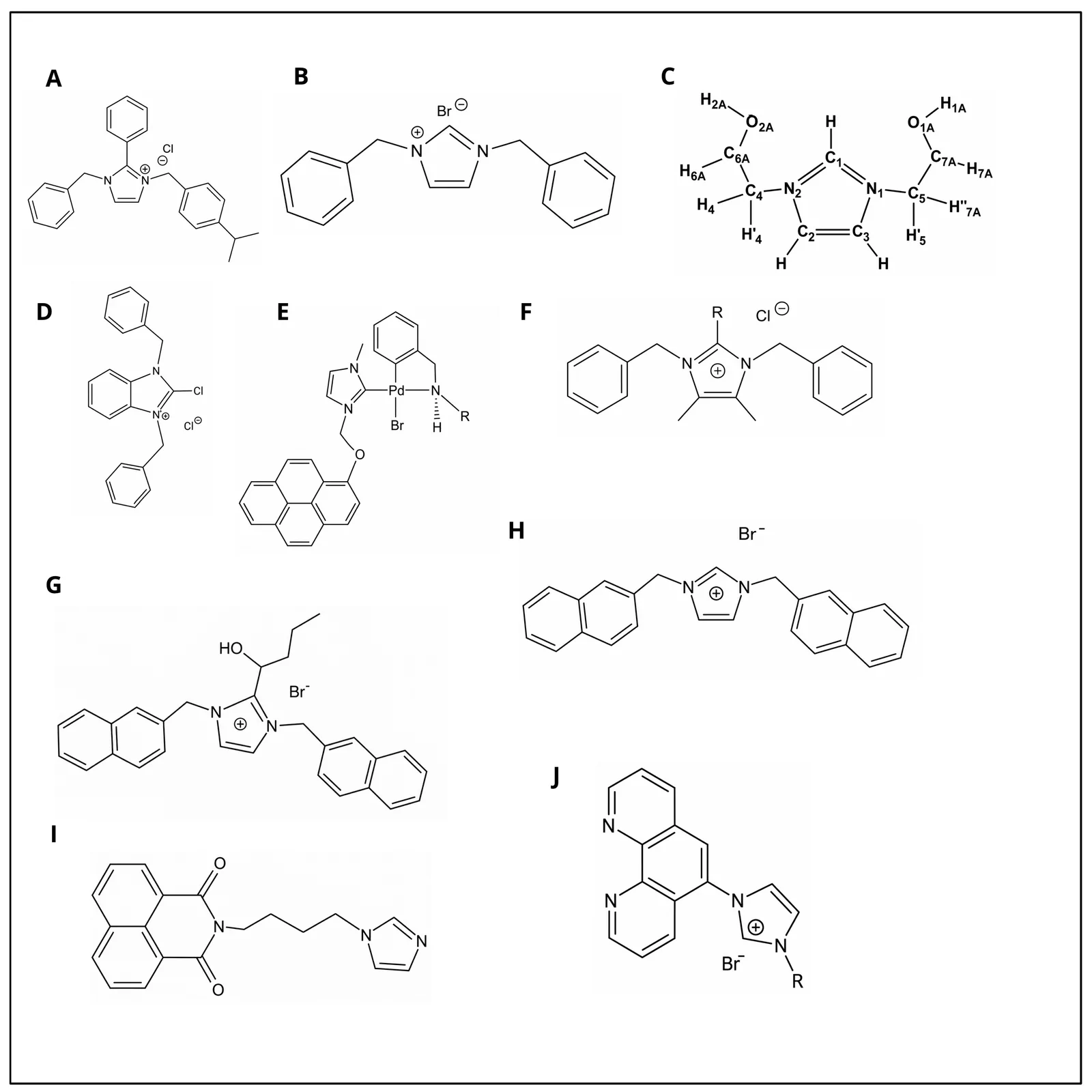
Chemical structures of IMSDs bearing aromatic and alkyl substituents described in Table 3. (**A**) 1-benzyl-2-phenyl-3-(4-isopropyl)-benzyl-imidazolium chloride), (**B**) 1,3-bisbenzylimidazolium bromide, (**C**) Imidazolium bromide derivatives: 3-(2-carboxyethyl)-1-(3-aminopropyl) 1H-imidazol-3-ium bromide), (**D**) 2-chloro-1,3-bis(4-nitrobenzyl)-1H-benzo[d]imidazole-3-iumchloride, (**E**) Imidazolium salt 1(H)Br derivatives, (**F**) 1,3-dibenzylmethylimidazolium chloride derivatives, (**G**) 2-(1-hydroxypentyl)-1,3-bis(naphthalen-2-ylmethyl)-imidazolium bromide, (**H**) 1,3-bis(naphthalen-2-ylmethyl)-imidazolium bromide, (**I**) Radioactively with radioactive iodine 131I 1,8- naphthalene monoimide bearing imidazolium salt, (**J**) 1-bromo-4-(dodecyloxy)-imidazolium bromide.

IMSDs bearing aromatic and alkyl substituents represent a structurally diverse class of cationic compounds with broad biological activity, including pronounced anticancer effects. A comprehensive evaluation of their cytotoxic properties has been performed using a range of tumor cell lines. These studies demonstrated that subtle structural modifications, including aromatic substitution, alkyl chain length, and the introduction of additional functional groups, can markedly influence their biological activity. The following section summarizes the most significant findings reported between 2003 and 2024, highlighting how molecular design affects the mechanism of action and anticancer potency of these structurally modified IMSDs. The structural formulas of the discussed compounds are presented in Figure 4A–J, while Table 3 summarizes the structurally modified imidazolium salt derivatives evaluated for their anticancer properties. This overview provides a comparative analysis of their activity against various tumor cell lines, indicating their anticancer efficacy and the principal biological pathways underlying their effects.

### 4.1. Imidazolium Bromide Derivatives

The 1,3-bisbenzylimidazolium bromide (IBN) derivatives are among the most thoroughly investigated compounds in this class. The lead compound, IBN-1, demonstrated potent cytotoxic activity against hepatocellular carcinoma cell lines including Hep3B and HepG2, with IC_50_ values of 5.5 μM and 4 μM, respectively. The present study revealed that these compounds induce apoptosis through inhibition of the EGFR/Ras/MEK/ERK signaling pathway. Furthermore, the compound suppresses survivin expression and modulates cell cycle–related proteins, thereby contributing to the inhibition of tumor cell proliferation [51,52].

Similarly, IBN-9 (1-Mesityl-3-(4-acetate-benzyl)-imidazolium chloride) exhibited moderate activity against HeLa cells (IC_50_ = 120 μM), which was associated with downregulation of survivin levels and cyclin D1 inhibition leading to cell cycle arrest [52].

Other imidazolium bromide derivatives bearing carboxyethyl or aminopropyl substituents, exhibited moderate activity. Among them 3-(2-carboxyethyl)-1-(3-aminopropyl)-1H-imidazol-3-ium bromide demonstrated the highest activity, with an IC_50_ = 57 μM [53]. Although the introduction of polar substituents aqueous solubility, it was accompanied by a moderate reduction in cytotoxic potency compared with the aromatic benzyl analogues.

### 4.2. Benzimidazolium and Substituted Aromatic Derivatives

Replacement of the imidazole core with a benzimidazole scaffold resulted in the development of highly active analogues. Among them, 2-chloro-1,3-bis(4-nitrobenzyl)-1H-benzo[d]imidazole-3-ium chloride exhibited potent cytotoxic activity, with an IC_50_ = 8.6 μM across several carcinoma cell models [54].

The introduction of electron-withdrawing substituents, such as nitro and chloro groups, increased the electrophilic character of the molecule, thereby enhancing its interactions with DNA and cellular proteins. These structural modifications also contributed to cell-cycle arrest and apoptosis induction, often via suppression of cyclin D1 expression [52].

### 4.3. Naphthalene–Imidazolium Hybrids

A structurally distinct group of derivatives combines naphthalene or naphthalimide aromatic systems with imidazolium cations. Compounds such as 1,3-bis(naphthalen-2-ylmethyl)-imidazolium bromide and its 2-(1-hydroxypentyl) analogues exhibited potent cytotoxic activity against lung cancer cell lines (H460, H1975 and HCC827), with IC_50_ values ranging from 3 to 9 μM [55,56]. Their anticancer activity has been attributed to the planar aromatic naphthalene moieties, which facilitate DNA intercalation and disrupt DNA replication, while the positively charged imidazolium centre enhances electrostatic interactions with nucleic acids and negatively charged cellular membranes [55,56].

IMSDs incorporating a 1,8-naphthalene monoimide (NMI)-moiety represent a significant advance in the development of hybrid anticancer agents. These compounds exhibited potent cytotoxic activity against colon (CaCo-2), prostate (PC-3), and breast (MCF-7) cancer cells with IC_50_ values ranging from approximately 0.04 to 20 μM [57].

Moreover, radiolabeled analogues, such as 131I-NMI demonstrated approximately seven-fold higher uptake in MCF-7 breast cancer cells compared to normal HEK-293 cells, highlighting their potential for targeted radiotheranostic applications [57].

### 4.4. Alkoxy-Substituted Imidazolium Halides

Recent studies have also focused on alkoxy-substituted imidazolium halides, including 1-bromo-4-(dodecyloxy)-imidazolium bromide and 1-bromo-4-(octyloxy)-imidazolium bromide. These amphiphilic cations contain long alkyl chains that enhance membrane affinity and facilitate cellular uptake [58].

These compounds exhibited moderate cytotoxic activity, with IC_50_ values ranging from 12.8 to 17.8 μM. Their anticancer effects are associated with the induction of apoptosis and the downregulation of Bcl-2 expression, suggesting the mitochondrial apoptotic pathway. This class of compounds represents an emerging group of lipid-mimetic imidazolium salts designed to exploit the unique physicochemical properties of cancer cell membranes [58].

Despite their structural diversity, imidazolium and benzimidazolium derivatives share several common mechanisms of action. Most compounds exert their anticancer effects by inducing apoptosis, often accompanied by inhibition of proliferative signaling pathways, suppression of DNA synthesis, and cell cycle arrest [43,47,49,58].

Derivatives containing aromatic or extended conjugated systems generally exhibited greater cytotoxic potency, most likely owing to enhanced π–π stacking interactions with DNA and improved membrane permeability. In contrast, compound lacking these structural features typically displayed lower activity or selective cytotoxicity toward specific cancer cell lines [59].

Electron-withdrawing substituents, such as nitro or chloro groups, further enhanced cytotoxic activity by increasing the electrophilic character of the molecules [60]. Conversely hydrophobic alkyl and aryl substituents improved cellular uptake through stronger interactions with lipid membranes [61]. The derivatives investigated combine hydrophilic functional groups that enhance aqueous solubility with hydrophobic aromatic moieties that promote target binding, potentially contributing to favorable amphiphilic balance and improved anticancer activity.

As presented in Table 3, structurally modified imidazolium salts bearing aromatic and alkyl substituents exhibit promising anticancer activity against a variety of cancer cell lines. Their cytotoxic effects have been linked to multiple mechanisms of action, including apoptosis induction through modulation of intracellular signaling pathways, inhibition of cell cycle progression, suppression of DNA synthesis, and regulation of apoptosis-related proteins. For example, 1-benzyl-2-phenyl-3-(4-isopropyl)benzyl-imidazolium chloride inhibited the EGFR/Raf/MEK/ERK signaling pathway and induced apoptosis through activation of caspase-9, caspase-3, and PARP cleavage in Hep3B cells, thereby suppressing tumor cell proliferation [51,52]. Similarly, selected imidazolium bromide derivatives promoted apoptosis by modulating the expression of Bcl family proteins, including increased Bax expression, resulting in enhanced apoptotic cell death in MCF-7 breast cancer cells [58].

**Table 3:** Structurally modified IMSDs bearing aromatic and alkyl substituents: structural influence on antitumor activity.

Type of Cancer	Experimental Model/ Cell Lines	IMSDs	IC_50_/ED_50_/Results/Mechanism			IC_50_ of Reference Drug	Figure/Ref.
Hepatic cancer	Hep3B	1-benzyl-2-phenyl-3-(4-isopropyl) benzyl-imidazolium chloride)	Cytotoxic effect IC_50_ = 5.5 μM	Inhibition of cell proliferation (MTS assay), induced apoptosis and inhibiting EGRF/Raf/MEK/ERK signaling, activation of caspase-9, caspase-3 and PARP cleavage (Western blot assay)		Sorafenib IC_50_ = 5 μM	Figure 4A,B [51,52]
	HepG2	1,3-bisbenzylimidazolium bromide	Cytotoxic effect IC_50_ = 4 μM				
		Imidazolium bromide derivatives: 3-(2-carboxyethyl)-1-(3-aminopropyl) 1H-imidazol-3-ium bromide)	Cytotoxic effect IC_50_ = 57 μM Inhibition of cell proliferation (MTT assay)			ND	Figure 4C [53]
		2-chloro-1,3-bis(4- nitrobenzyl)-1H-benzo]imidazole-3-ium chloride	Cytotoxic effect IC_50_ = 8.6 μM Inhibition of cell proliferation (MTT assay)			Cisplatin IC_50_ = 9.41 μM	Figure 4D [54]
	HLE	(1-Mesityl-3-(4-acetate-benzyl)-imidazolium chloride)	Cytotoxic effect IC_50_ = 120 μM Decrease in survivine concentrations inhibit cyclin D1 (Western blot assays)			5-fluorouracul IC_50_ approximately twofold lower than tested agents	Figure 4A,B [51]
Cervical cancer	HeLa	Imidazolium bromide derivatives: 3-(2-carboxyethyl)-1-(3-aminopropyl) 1H-imidazol-3-ium bromide)	Cytotoxic effect IC_50_ = 81 μM Inhibition of cell proliferation (MTT assay)			ND	Figure 4C [53]
		Imidazolium salt 1(H)Br derivatives	Cytotoxic effect IC_50_ = 3.7–5.9 μM Inhibition of cell proliferation (MTT assay), inhibiting DNA synthesis (DNA melting assay)			Cisplatin IC_50_ = 26.19 μM	Figure 4E [62]
Lung carcinoma	A-549	1,3-dibenzylmethylimidazolium chloride derivatives	Cytotoxic effect ED_50_ > 10 μg/mL (The cytotoxicity assay was not specified)			ND	Figure 4F [59]
Lung carcinoma	A-549	2-chloro-1,3-bis(4- nitrobenzyl)-1H-benzo[d]imidazole-3-ium chloride	Cytotoxic effect IC_50_ = 8.6 μM Inhibition of cell proliferation (MTT assay)			Cisplatin IC_50_ = 9. 57M	Figure 4D [54]
		2-(1-hydroxypentyl)-1,3-bis(naphthalen-2-ylmethyl)-imidazolium bromide	Cytotoxic effect IC_50_ = 9 μM		Inhibition of cell proliferation (MTT assay)	Cisplatin IC_50_ = 5 μM	Figure 4G [55]
	H460		Cytotoxic effect IC_50_ = 7 μM			Cisplatin IC_50_ = 3 μM	
		1,3-bis(naphthalen-2-ylmethyl)-imidazolium bromide	Cytotoxic effect IC_50_ = 4 μM		Inhibition of cell proliferation (MTT assay), PARP-1 cleavage and reduction in procaspase-3 (Western blot assays)	Cisplatin IC_50_ = 3 μM	Figure 4H [56]
	HCC827		Cytotoxic effect IC_50_ = 9 μM			Cisplatin IC_50_ = 4 μM	
		2-(1-hydroxypentyl)-1,3-bis(naphthalen-2-ylmethyl)-imidazolium bromide	Cytotoxic effect IC_50_ = 5 μM		Inhibition of cell proliferation (MTT assay)	Cisplatin IC_50_ = 3 μM	Figure 4G [55]
	H1975		Cytotoxic effect IC_50_ = 3 μM			Cisplatin IC_50_ = 10 μM	
	H1975	1,3-bis(naphthalen-2-ylmethyl)-imidazolium bromide	Cytotoxic effect IC_50_ = 6 μM Inhibition of cell proliferation (MTT assay), PARP-1 cleavage and reduction in procaspase-3 (Western blot assays)			Cisplatin IC_50_ = 10 μM	Figure 4H [56]
Bladder carcinoma	UMUC3	1,3-dibenzylmethylimidazolium chloride derivatives	Cytotoxic effect ED_50_ = 6.5 mg/mL (The cytotoxicity assay was not specified)			ND	Figure 4F [59]
Colon adenocarcinoma	HT-29		Cytotoxic effect ED_50_ 1 >10 μg/mL (The cytotoxicity assay was not specified)				
	CaCo-2	Radioactively with radioactive iodine 131I 1,8- naphthalene monoimide bearing imidazolium salt (NMI)	Cytotoxic effect IC_50_ = 10 Mm (Inhibition of cell proliferation (MTT assay)				Figure 4I [57]
Prostate cancer	PC-3		Cytotoxic effect IC_50_ = 0.04 μM (Inhibition of cell proliferation (MTT assay),				
		1,3-dibenzylmethylimidazolium chloride derivatives	Cytotoxic effect ED_50_ > 10 μg/mL				Figure 4F [59]
Pancreatic adenocarcinoma	PACA2	1,3-dibenzylmethylimidazolium chloride derivatives	Cytotoxic effect ED_50_ = 1.38–10.0 µg/mL			ND	Figure 4F [59]
Kidney carcinoma	A498_2_LM		Compounds were inactive against the cell line. ED_50_ >10 μg/mL				
Breast carcinoma	MCF-7	Imidazolium salt 1(H)Br derivatives	Cytotoxic effect IC_50_ = 0.01–3.2 μM Inhibiting DNA synthesis Inhibition of cell proliferation (MTT assay), inhibiting DNA synthesis (DNA melting assay)			Cisplatin IC_50_ = 24.85 μM	Figure 4E [62]
		Radioactively with radioactive iodine 131I 1,8- naphthalene monoimide bearing imidazolium salt	Cytotoxic effect IC_50_ = 10 mM (Inhibition of cell proliferation (MTT assay),			ND	Figure 4I [57]
		1-bromo-4-(dodecyloxy)-imidazolium bromide	Cytotoxic effect IC_50_ = 15.4 μM		Inhibition of cell proliferation (MTT assay). Induce apoptosis, Bcl-inhibitor, Bax activation (Western blot assay)	Doxorubicin IC_50_ = 5.2 μM Cisplatin IC_50_ = 20.7 μM	Figure 4J [58]
	MDA-MB-231		Cytotoxic effect IC_50_ = 12.8 μM			Doxorubicin IC_50_ = 1.07 μM Cisplatin IC_50_ = 21.5 μM	
			Cytotoxic effect IC_50_ = 17.8 μM				
		1,3-dibenzylmethylimidazolium chloride derivatives	Cytotoxic effect ED_50_ = 1.66 mg/mL			ND	Figure 4F [59]
Ovarian carcinoma	FDIGROV		Cytotoxic effect ED_50_ = 5.3–7.4 μg/mL				

IC_50_ (half maximal inhibitory concentration), ED_50_ (Effective Dose 50%), ND not determined.

In addition to the induction of apoptosis, several compounds interfered with cell cycle progression and DNA replication. Treatment of HLE hepatocellular carcinoma cells with 1-mesityl-3-(4-acetatebenzyl)-imidazolium chloride resulted in decreased survivin expression together with inhibition of cyclin D1, suggesting cell cycle arrest and reduced proliferative capacity [51]. Furthermore, imidazolium salt 1(H)Br derivatives inhibited DNA synthesis in HeLa and MCF-7 cells, which contributed to the suppression of cancer cell proliferation. Other imidazolium derivatives induced apoptosis through PARP-1 cleavage and reduction in procaspase-3 levels in H460 and H1975 lung cancer cells, further supporting the involvement of caspase-dependent apoptotic pathways [56].

Overall, many of the investigated imidazolium complexes exhibited potent anticancer activity, and in several experimental models their IC_50_ values were comparable to or lower than those of the reference drugs, indicating high cytotoxic efficacy at relatively low concentrations. For example, 2-chloro-1,3-bis(4-nitrobenzyl)-1H-benzo[d]imidazole-3-ium chloride showed slightly greater activity than cisplatin against HepG2 and A549 cells [54], whereas imidazolium salt 1(H)Br derivatives were considerably more potent than cisplatin in MCF-7 breast cancer cells. Likewise, the ^131^I-labelled 1,8-naphthalene monoimide bearing an imidazolium moiety demonstrated remarkable activity against PC-3 prostate cancer cells (IC_50_ = 0.04 μM) [57]. However, the cytotoxic activity was strongly dependent on both the chemical structure and the cancer cell type. In particular, several bromide-containing imidazolium derivatives evaluated against A549, H460, and HCC827 lung cancer cells exhibited higher IC_50_ values than cisplatin, indicating lower cytotoxic potency [55,56]. Taken together, these findings demonstrate that structural modification of imidazolium salts with aromatic and alkyl substituents can substantially influence their anticancer activity and mechanism of action, supporting further investigation of these compounds as promising anticancer agents.

## 5. Cytotoxicity of IMSDs in Non-Tumor Cell Lines

Several imidazolium-based compounds and their derivatives have been studied for their anticancer potential, with particular emphasis on their ability to selectively target cancer cells toward normal tissues (Table 4). One of the extensively studied compounds,1-Benzyl-2-phenyl-3-(4-isopropyl)-benzyl-imidazolium chloride (IBN-65), demonstrated activity against hepatocellular carcinoma while exhibiting no detectable antiproliferative effects toward normal liver (THLE-2), breast (MCF-10), and lung fibroblast (IMR90) cell lines, suggesting preferential activity toward tumor cells [51,52]. Similarly, a 1,8-naphthalene monoimide bearing an imidazolium salt) displayed significantly lower cytotoxicity toward non-cancerous HEK-293 kidney cells (IC_50_ = 35 µM) than toward MCF-7 and PC-3 cancer cells lines suggesting preferential toxicity toward malignant cells [57].

Among metal-based derivatives, Pt(II)-NHC complexes exhibited variable cytotoxicity in normal human cell lines. Platinum(II) complexes containing selenophene, thiophene or a benzimidazole-derived C^N^N scaffold showed IC_50_ values of 3.26–4.94 µM in CCD-19Lu lung fibroblasts, while maintaining favorable selectivity indices ranging from 15.52 to 164.67, considerably exceeding those observed for cisplatin (IC_50_ = 38.21 µM) [29]. Likewise, Pt(II)-NHC complexes based on 4,5-diarylimidazole displayed IC_50_ values between 1.01 and 12.94 µM in LO2 normal liver cells, with SI values reaching 39.21, indicating pronounced selectivity toward cancer cells [32].

Fe-NHC complexes exhibited comparatively lower toxicity toward human dermal fibroblasts, with IC_50_ values ranging from 23.6 to 42.04 µM and selectivity indices between 7.24 and 48.32, suggesting a favorable safety profile [27]The biological activity of gold(I)-NHC complexes strongly depended on their chemical structure. Imidazolyl gold(I) compounds containing triphenylphosphine as a co-ligand exhibited low toxicity toward IMR90 lung fibroblasts (IC_50_ = 63–87 µM) with SI values ranging from 0.88 to 48.30 [30]. In another study, Au(4,5-dichloro, *N*-methyl, *N′*-[2-hydroxyethylphenyl]imidazol-2-ylide) maintained approximately 70% viability of HaCaT keratinocytes at 5 µM, whereas cisplatin preserved more than 80% viability after 24 h exposure, confirming relatively low toxicity toward normal skin cells [33].

Silver-NHC complexes also demonstrated acceptable safety profiles. Ag(I)-NHC complexes derived from 1,3-dioxane ligands showed IC_50_ values of 11.09–36.38 µM in L-929 fibroblasts with SI values ranging from 1.36 to 11.33, whereas silver biscarbene and monocarbene complexes exhibited IC_50_ values of 10.7–26.3 µM and 10.3–12.2 µM, respectively, in Vero-E6 kidney epithelial cells. Their selectivity indices ranged from 0.38 to 3.50 for biscarbene derivatives and 0.46 to 1.23 for monocarbene analogues, indicating moderate selectivity depending on the structure [25,26].

Lithocholic acid (LCA)-based imidazolium salts exhibited consistently low toxicity toward several normal cell models. In CRL-1475 human fibroblasts, most compounds displayed IC_50_ values exceeding 100 µg/mL, except for the propyl derivative (S3), with an IC_50_ of 36.5 µg/mL. Their selectivity indices ranged from 0.64 to 5.54, with the dodecyl (S9) and hexadecyl (S10) derivatives showing the highest selectivity toward colon cancer cells [45,63]. In THP-1 human monocytes, IC_50_ values exceeded 77 µg/mL, while SI values ranged from 0.54 to 14.67, indicating generally low toxicity and favorable selectivity for several derivatives. More recently, these IMSDs also demonstrated low toxicity toward H9C2 cardiomyocytes, with IC_50_ values between 52.09 and 105.4 µg/mL and SI values ranging from 0.75 to 3.99, comparable to or exceeding the safety profile of doxorubicin (IC_50_ = 51.71 µg/mL) [46].

Low toxicity toward healthy cells was also confirmed for 2-chloro-1,3-bis(4-nitrobenzyl)-1H-benzoimidazol-3-ium chloride, which maintained high viability of mouse embryonic fibroblasts (MEF) [53]. Furthermore, recently developed halogen-free metal-phenolic imidazolium poly(ionic liquids) (PVD@Zn) not only exhibited excellent biocompatibility but also enhanced L929 fibroblast proliferation by approximately 22%, suggesting their ability to actively promote tissue regeneration rather than merely avoiding cytotoxicity [64].

The imidazolium-based compounds and N-heterocyclic carbene derivatives generally possess favorable safety profiles toward non-cancerous cells while maintaining potent anticancer activity. Importantly, numerous compounds exhibit high selectivity indices, particularly platinum-, iron-, and lithocholic acid-based derivatives, supporting their continued development as selective anticancer agents with reduced off-target toxicity.

Studies on IMSDs and N-heterocyclic carbene (NHC) ligands suggest that many of these molecules exhibit a high level of selectivity toward cancer cells while showing minimal toxicity toward healthy cells. Although some derivatives, particularly platinum and silver complexes, showed moderate toxicity in non-tumor cells, the majority of tested compounds demonstrated low toxicity in normal tissue models [25,29]. On the other hand, iron- and gold-based complexes exhibited high viability in normal cell lines (THLE-2, MCF-10, IMR90, and HaCaT). The IC_50_ values for these healthy cells were not determined, whereas IC_50_ values for cancer cells were significantly lower, below 10 µM, further confirming the strong selectivity of these compounds [51,52]. In the case of imidazole complexes with lithocholic acid, containing 6–8 carbon atoms in the alkyl chain, the cytotoxic activity against cancer cells (DLD-1, MCF-7 and MDA-MB-231) was significantly stronger, whilst the viability of healthy cells remained significantly higher [46,63]. It should also be noted that, in the majority of the cited studies, the analyzed imidazolium complexes showed a favorable cytotoxicity profile toward healthy cells compared with commonly used reference drugs. These findings may indicate a greater selectivity of the investigated compounds toward cancer cells, which represents an important feature from the perspective of potential therapeutic applications and the reduction in adverse effects associated with chemotherapy. An exception was observed for platinum-based imidazolium complexes, which demonstrated significantly increased cytotoxicity toward lung fibroblasts [29], suggesting the possibility of side effects related to their impact on healthy tissues. Nevertheless, most of the analyzed complexes were characterized by a favorable biological activity profile, combining high anticancer efficacy with a limited effect on normal cells.

These findings highlight the potential of imidazolium- and NHC-based compounds as selective and relatively safe candidates for the further development of anticancer drugs.

## 6. Antitumor Efficacy and Safety of IMSDs in In Vivo Models

IMSDs have demonstrated promising antitumor activity and acceptable safety profiles in a variety of in vivo cancer models, supporting their potential as selective and effective anticancer agents. The available in vivo studies investigating the anticancer activity of IMSDs are summarized in Table 5.

The earliest in vivo study investigating the anticancer potential of IMSDs evaluated a silver(I) N-heterocyclic carbene complex derived from 4,5-dichloro-1H-imidazole in a murine ovarian cancer (OVCAR-3) xenograft model. Histopathological examination demonstrated extensive tumor necrosis and cancer cell death, while no adverse effects were observed in the brain, liver, lungs, heart, kidneys, or spleen, indicating selective cytotoxicity with minimal systemic toxicity [34].

In a non-small cell lung cancer (A549) xenograft model, 1-mesityl-3-(2-naphthoylmethano)-1H-imidazolium bromide (MNIB) was administered intraperitoneally at doses ranging from 10 to 80 mg/kg. Treatment resulted in a statistically significant reduction in tumor volume (*p* < 0.05–0.001) compared with the control group. Importantly, no acute toxicity was detected at any tested dose, demonstrating a favorable safety profile [65].

The antitumor efficacy of several imidazolium salts was further investigated in hepatocellular carcinoma (Huh7) xenografts. Treatment with IBN-65 (5 mg/kg, intraperitoneally, once weekly for four weeks) reduced tumor volume by more than 65%, inhibited angiogenesis through decreased CD31 expression, and reduced intratumoral fibrotic tissue without causing significant systemic toxicity or body weight loss [52]. In contrast, oral administration of IBN-1 (2 g/L in drinking water) produced only a 31% reduction in tumor size and inhibited survivin expression but was associated with approximately 9% body weight loss, indicating increased systemic toxicity [51]. Another compound, IBN-9, administered orally in drinking water (0.6 or 1.5 g/L), inhibited the expression of Ki67, survivin, and Cdk4 and reduced tumor size by approximately 45% and 60% at the lower and higher doses, respectively, without affecting body weight [51,52].

In an induced mouse model of bladder cancer, the triphenylphosphonium-containing imidazolium salt TPP1 was administered intravesically at concentrations of 1500 or 3000 μM. Immunohistochemical analysis revealed increased activation of caspase-3 and extensive tumor necrosis, demonstrating effective induction of apoptosis while supporting the potential of TPP1 as a localized therapeutic agent [66].

Zebrafish (*Danio rerio*) models were used to evaluate iron-based IMS complexes in colorectal cancer (HCT116). The cationic complex exhibited lower toxicity (LD50 = 24.624 μM) and reduced tumor cell proliferation by approximately 20%, whereas the neutral complex was more toxic (LD50 = 6.861 μM) but demonstrated greater antiproliferative activity, reducing tumor cell proliferation by approximately 50% [27].

**Figure 5:**
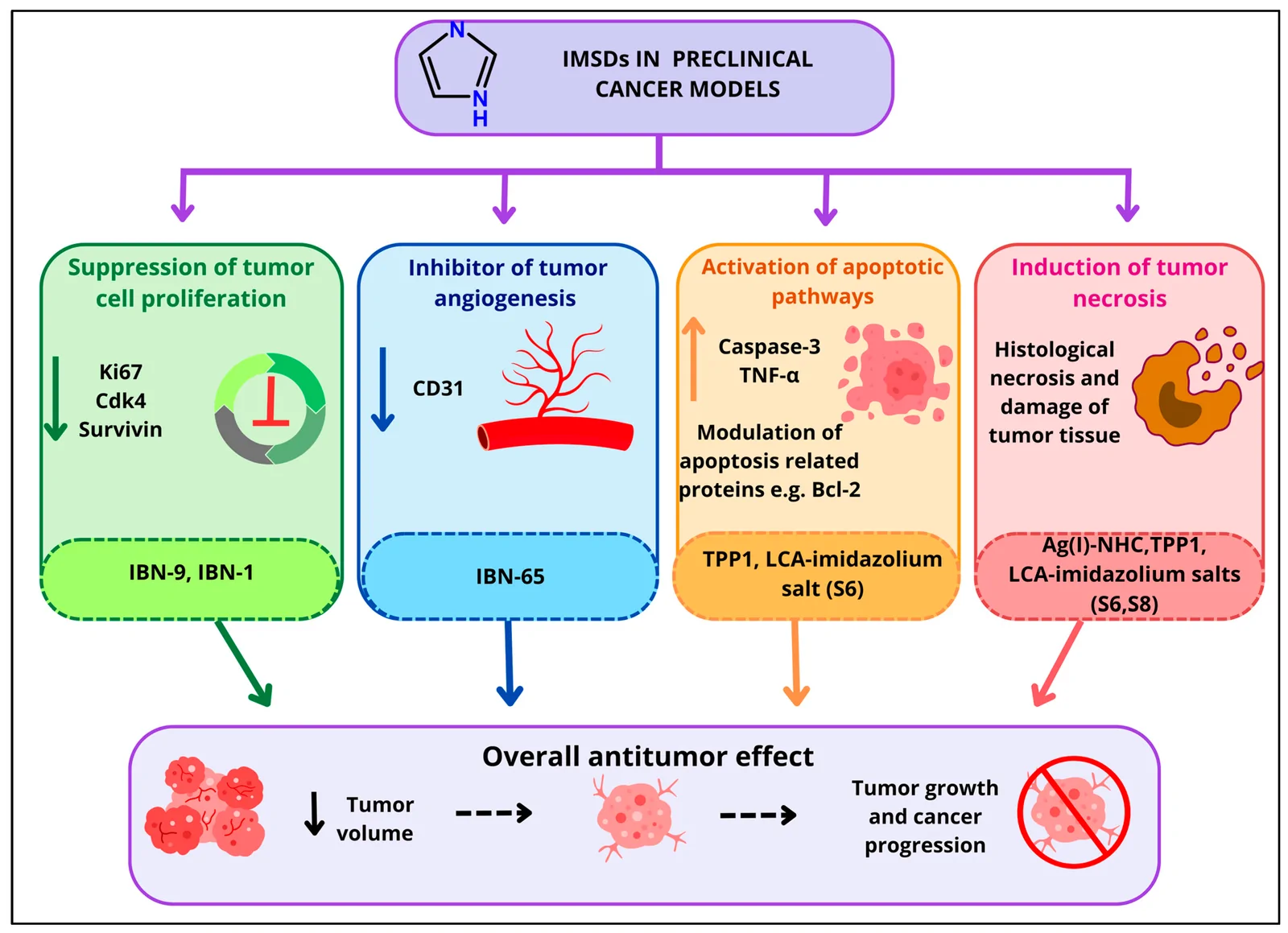
The main mechanisms of action of IMSDs in preclinical cancer models.

In a colorectal adenocarcinoma (DLD-1) xenograft model, an LCA-based imidazolium salt containing a six-carbon alkyl chain (S6) was administered orally at doses of 100–500 mg/kg. At the highest dose, treatment significantly decreased TNF-α levels, increased Bcl-2 and caspase-3 expression, and induced marked tumor necrosis (80.2%) while reducing Ki67 expression by approximately 60%. Hematological analysis and gross observations revealed no evidence of toxicity, and no significant body weight changes were observed. Notably, the compound demonstrated greater antitumor efficacy than 5-fluorouracil (5-FU) [45]. More recently, an LCA-based imidazolium salt containing an eight-carbon alkyl chain (S8) was evaluated in an MCF-7 breast cancer xenograft model. Oral administration (300–500 mg/kg) significantly reduced tumor volume compared with both the untreated control and doxorubicin-treated groups. Histopathological analysis revealed more than 60% tumor necrosis, while gross examination of organs showed no signs of systemic toxicity, further supporting the therapeutic potential of this class of compounds [46]. The available in vivo data demonstrate that imidazolium salts and NHC-based compounds exhibit potent antitumor activity while maintaining generally acceptable safety profiles in preclinical animal models. Their anticancer effects are mediated through multiple complementary mechanisms, including the induction of apoptosis, inhibition of angiogenesis, suppression of tumor cell proliferation, and modulation of proteins involved in cell survival. The principal mechanisms of action of imidazolium-based salts in preclinical cancer models, are summarized in Figure 5. One of the major mechanisms involves inhibition of tumor cell proliferation through the downregulation of the expression of cell cycle regulators such as Ki-67 and Cdk4 [51,52]. In addition, IMSDs suppress tumor angiogenesis, as demonstrated by decreased CD31 expression, which curtails the formation of new blood vessels and consequently restricts the supply of oxygen and nutrients to the tumor tissue [52]. Another important mechanism is the induction of tumor cell death, manifested by necrosis and marked reduction in tumor volume in treated animals. Furthermore, IMSDs activate apoptotic pathways by modulating apoptotic-related mediators, including, caspase-3, Bcl-2 and TNF-α resulting in enhanced cancer cell death [45,46]. Collectively, these findings demonstrate that imidazolium-based salts exert their antitumor effects through multiple complementary mechanisms that simultaneously inhibit tumor growth, impair angiogenesis, and promote both apoptotic and necrotic cell death.

Despite these promising findings, the available in vivo evidence remains limited. Most studies were performed using relatively small numbers of animals per experimental group, which may reduce the statistical power and reproducibility of the reported results. Moreover, comprehensive pharmacokinetic studies, including absorption, distribution, metabolism, and excretion (ADME), are largely lacking, making it difficult to predict systemic exposure and optimal dosing regimens. Similarly, detailed toxicological evaluations, particularly long-term and repeated-dose toxicity studies, have not been performed for most compounds. In addition, only a limited number of tumor models has been investigated, and direct comparisons with clinically approved anticancer agents are scarce. Future studies should therefore include standardized efficacy protocols, comprehensive pharmacokinetic and toxicological assessments, larger animal cohorts, and validation across multiple tumor models to better establish the translational potential of imidazolium-based salts as anticancer agents.

## 7. Structure–Activity Relationships and the Most Promising Classes of IMSDs

**Figure 6:**
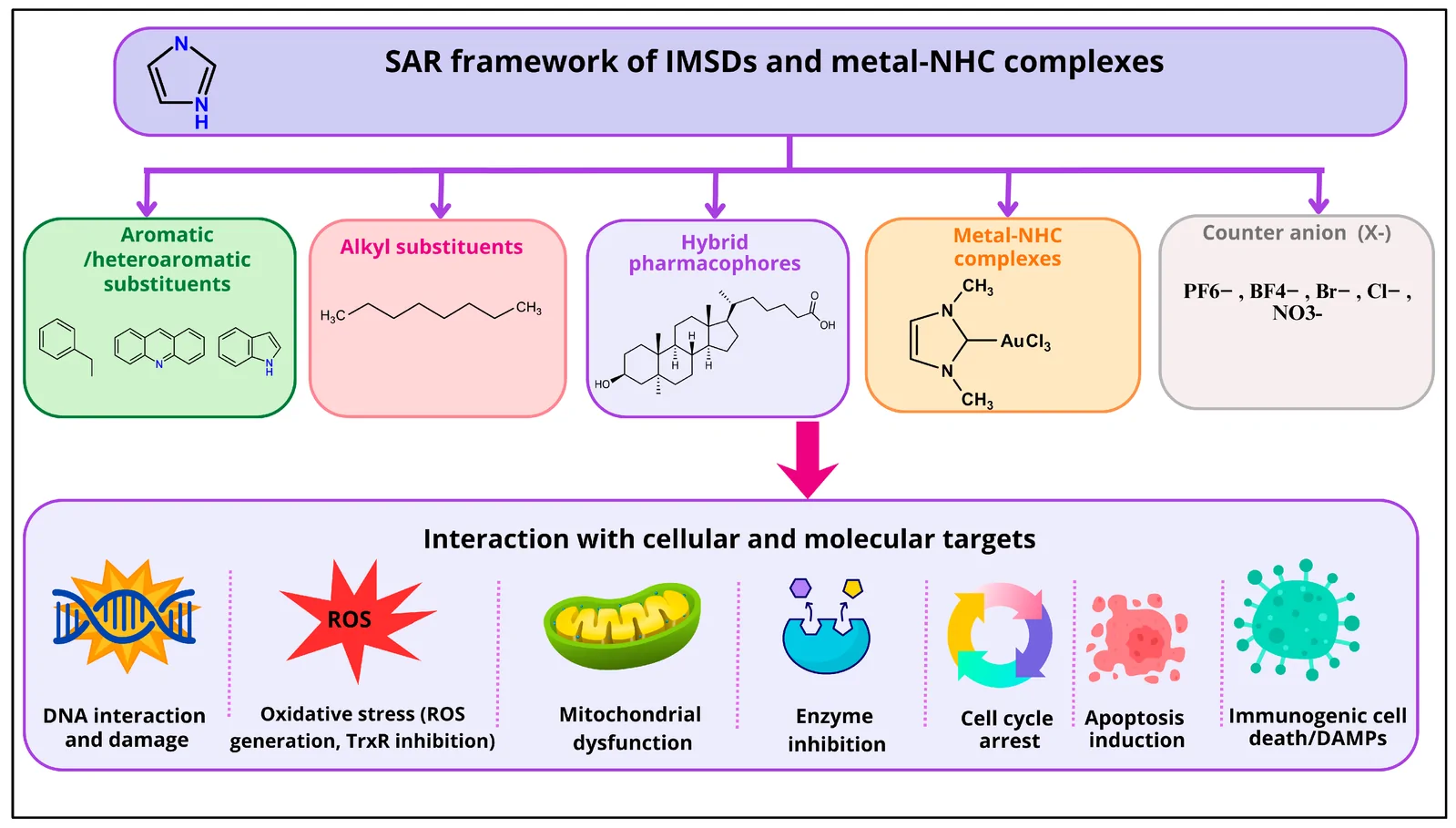
SAR framework and antitumor mechanisms of IMSDs and metal-NHC complexes.

Despite the considerable structural diversity of the imidazolium salt derivatives (IMSDs) discussed in this review, SAR trends can be identified (Figure 6/Table 6). The available evidence indicates that both the imidazolium scaffold and the nature of the substituents attached to the heterocyclic core play pivotal roles in determining cytotoxic potency, selectivity, and the underlying mechanisms of anticancer activity. Although direct comparison between individual compounds is complicated by differences in experimental models, cancer cell lines, and biological assays, analysis according to structural class reveals recurring patterns that provide valuable insight into the molecular features associated with enhanced therapeutic potential.

### 7.1. Aromatic and Heteroaromatic Substituents

One of the most consistent SAR observations concerns the beneficial effect of aromatic and heteroaromatic substituents attached to the imidazolium scaffold. The introduction of bulky aromatic groups, including naphthalene, acridine, indole, quinoline, or benzimidazole moieties, was repeatedly associated with enhanced antiproliferative activity across different cancer models [41,42,48,50,51,52,54,55,58].

The improved biological activity of these derivatives is most likely associated with increased π-conjugation and lipophilicity, which facilitate cellular uptake and strengthen interactions with intracellular targets such as DNA and regulatory proteins. These structural modifications also promote apoptosis, mitochondrial dysfunction, and inhibition of cancer cell proliferation. In several studies, aromatic derivatives demonstrated substantially lower IC_50_ values than structurally simpler analogues.

Despite these advantages, excessive structural complexity may adversely affect physicochemical properties. Highly lipophilic or sterically demanding aromatic systems may display reduced aqueous solubility or impaired bioavailability, indicating that careful optimization of aromatic substitution is necessary to achieve an appropriate balance between potency and drug-like properties.

### 7.2. Alkyl Substituents

The influence of alkyl substituents differs from that of aromatic groups and is primarily related to modulation of physicochemical properties rather than direct interaction with biological targets. Increasing alkyl chain length generally enhances lipophilicity and membrane permeability, facilitating intracellular accumulation of imidazolium derivatives. Moderate alkyl chains were frequently associated with improved cytotoxic activity, particularly among silver-containing complexes and lithocholic acid derivatives.

However, the relationship between alkyl chain length and biological activity is not linear. Several studies demonstrated that excessive elongation of the alkyl chain resulted in decreased selectivity and increased nonspecific cytotoxicity, probably due to excessive membrane disruption and reduced aqueous solubility. These findings suggest that optimization of alkyl substituents should focus on achieving an appropriate balance between membrane permeability and selective toxicity toward malignant cells rather than maximizing hydrophobicity [43,48,49].

### 7.3. Hybrid Imidazolium Derivatives

Hybrid imidazolium derivatives have also been investigated as potential anticancer agents because they combine the physicochemical properties of the imidazolium scaffold with the biological activity of established pharmacophores. The incorporation of moieties such as curcumin, chalcone, acridine, quinoline, indole, ferrocene, or lithocholic acid frequently resulted in compounds capable of simultaneously modulating several molecular targets involved in cancer progression [44,45,46].

Unlike simple imidazolium salts, hybrid derivatives generally exhibit broader mechanisms of action, including DNA interaction, mitochondrial dysfunction, oxidative stress induction, apoptosis, and cell-cycle arrest. This structural strategy also offers greater flexibility for rational molecular optimization, allowing simultaneous improvement of lipophilicity, solubility, and tumor selectivity. Several hybrid compounds demonstrated enhanced selectivity indices compared with their parent structures, indicating that molecular hybridization represents an effective approach to improving the therapeutic profile of imidazolium derivatives [44,45,46].

However, hybridization does not always result in increased cytotoxic potency. In many studies, hybrid compounds exhibited higher IC_50_ values than the most active metal–NHC complexes, suggesting that the biological outcome depends strongly on the nature of the incorporated pharmacophore and the linker connecting individual fragments. Additional optimization is therefore required to identify the most favorable structural combinations.

### 7.4. Metal–NHC Complexes

Among all classes of IMSDs discussed in this review, metal–NHC complexes appear to represent promising candidates for further anticancer drug development. Their superior biological activity is primarily associated with the presence of a coordinated transition metal, which substantially expands the spectrum of biological targets and mechanisms of action. Gold(I), platinum(II), and silver(I) complexes consistently exhibited the lowest IC_50_ values reported in the literature, frequently reaching the submicromolar range, while maintaining activity against a broad panel of tumor cell lines, including drug-resistant models [20,21,22,23,24,25,26,27,28,29,30,31,32,33,34,35,37,38,39,40,64].

The enhanced activity of these complexes is largely attributed to their ability to simultaneously modulate multiple cellular pathways. Depending on the coordinated metal, they induce oxidative stress through reactive oxygen species generation, inhibit thioredoxin reductase, damage DNA, impair mitochondrial function, and activate intrinsic apoptotic signaling. Such multitarget activity reduces the likelihood that cancer cells can evade treatment through a single resistance mechanism. In addition, several studies demonstrated selective cytotoxicity toward malignant cells compared with normal fibroblasts or epithelial cells, suggesting a potentially favorable therapeutic window [20,21,22,23,24,25,26,27,28,29,30,31,32,33,34,35,37,38,39,40,64].

Nevertheless, despite these encouraging findings, several limitations remain. Most available studies have been performed exclusively in vitro, while comprehensive in vivo investigations are still scarce. Furthermore, systematic toxicological evaluation, pharmacokinetic characterization, and long-term safety studies are lacking for the majority of metal-containing derivatives. Consequently, although these compounds exhibit biological activity, their clinical potential cannot yet be fully assessed.

### 7.5. Structural Determinants of Selectivity and Safety

Although high cytotoxic potency remains one of the primary objectives in anticancer drug development, increasing evidence indicates that selectivity toward cancer cells is equally important. Several studies included in this review demonstrated that structural modifications designed to optimize lipophilicity, introduce hybrid pharmacophores, or coordinate specific transition metals may improve the therapeutic window by reducing toxicity toward normal cells. Particularly encouraging results were obtained for selected silver- and gold-containing complexes as well as several hybrid derivatives, which exhibited measurable selectivity indices or substantially higher IC_50_ values in normal fibroblasts than in tumor cells.

Nevertheless, assessment of safety remains one of the weakest aspects of the currently available literature. Only a limited number of studies evaluated normal cell lines or reported selectivity indices, and comprehensive in vivo toxicity studies remain largely unavailable. Consequently, although several structural modifications appear to improve tumor selectivity, the available evidence is still insufficient to identify universal structural determinants of safety. Future studies should therefore combine SAR optimization with systematic evaluation of pharmacokinetics, biodistribution, and toxicological profiles to facilitate the translation of these compounds into clinically relevant anticancer agents.

## 8. Current Limitations and Future Perspectives

Despite the encouraging in vitro and the growing number of in vivo studies demonstrating the anticancer potential of imidazolium salt derivatives (IMSDs), their translation into clinical oncology remains extremely limited. To date, none of the compounds discussed in this review has progressed to clinical trials specifically as an anticancer agent, highlighting the substantial gap between promising experimental findings and successful clinical development. This translational gap reflects several scientific and pharmacological challenges that should be addressed before IMSDs can be considered viable therapeutic candidates. One of the major limitations of the current literature is the overwhelming predominance of in vitro investigations. Although numerous derivatives exhibit submicromolar cytotoxicity against a wide spectrum of cancer cell lines, only a limited number have been evaluated in animal models. Consequently, the pharmacokinetic behavior, biodistribution, metabolic stability, and long-term therapeutic efficacy of most IMSDs remain largely unknown. Without comprehensive preclinical evaluation, it is difficult to predict whether the promising antiproliferative effects observed under laboratory conditions can be reproduced in vivo.

Another important limitation concerns safety assessment. Most published studies focus primarily on anticancer efficacy, whereas systematic toxicological investigations are scarce. Although several compounds demonstrated encouraging selectivity toward cancer cells, selectivity indices were reported only in a limited number of studies, and comprehensive evaluation of toxicity toward normal tissues, immunotoxicity, hepatotoxicity, nephrotoxicity, and genotoxicity is generally lacking. Future studies should therefore incorporate standardized toxicological protocols together with detailed pharmacokinetic analyses to establish the therapeutic window of these compounds [67]. Next area requiring considerable attention is the evaluation of combination therapy. Current studies have almost exclusively investigated IMSDs as single therapeutic agents, whereas their interactions with clinically established anticancer drugs remain largely unexplored. With the exception of a few reports demonstrating synergistic interactions with doxorubicin, systematic investigations involving platinum-based chemotherapy, taxanes, antimetabolites, targeted therapies, or immune checkpoint inhibitors are still lacking. Likewise, virtually no studies have examined the potential of IMSDs as radiosensitizers or their interactions with radiotherapy, despite the fact that several derivatives induce oxidative stress and DNA damage, mechanisms known to enhance radiation-induced cytotoxicity. These aspects represent particularly attractive directions for future translational research.

From a medicinal chemistry perspective, further structural optimization remains essential. Although current SAR studies indicate that metal coordination, bulky aromatic substituents, hybrid pharmacophores, and balanced lipophilicity generally improve biological activity, rational optimization has not yet been performed systematically. Future molecular design should focus not only on maximizing cytotoxic potency but also on improving selectivity toward malignant cells, aqueous solubility, metabolic stability, and pharmacokinetic properties while minimizing off-target toxicity.

The integration of imidazolium-based systems with nanomedicine and advanced drug delivery technologies represents an emerging area of research. Owing to their tunable amphiphilicity, ionic character, and structural versatility, imidazolium derivatives may serve not only as active anticancer agents but also as functional components of nanocarriers. Recent studies have demonstrated that imidazolium-based ionic liquids can improve drug solubility, loading efficiency, formulation stability, and stimulus-responsive drug release when incorporated into nanoparticles, liposomes, polymeric carriers, or mesoporous silica systems. Such multifunctional platforms may facilitate targeted drug delivery, reduce systemic toxicity, and improve accumulation within the tumor microenvironment through passive or active targeting strategies [68].

The convergence of imidazolium chemistry with nanotechnology may therefore represent one of the most promising future directions in this field. Beyond functioning as cytotoxic molecules, IMSDs could become structural components of smart drug-delivery systems capable of controlled drug release, co-delivery of multiple therapeutic agents, or surface functionalization with tumor-targeting ligands. Nevertheless, successful clinical translation of such multifunctional platforms will require standardized safety evaluation, scalable manufacturing procedures, regulatory harmonization, and comprehensive studies addressing long-term biocompatibility [67].

Overall, the future development of IMSDs should move beyond the synthesis of increasingly potent compounds toward a more comprehensive translational strategy that integrates rational molecular design, systematic toxicological evaluation, optimization of pharmacokinetic properties, investigation of combination therapies, and the development of targeted nanomedicine-based delivery systems. Addressing these challenges will be essential for transforming the considerable experimental potential of imidazolium salt derivatives into clinically relevant anticancer therapies.

## 9. Conclusions

IMSDs constitute a structurally diverse and rapidly evolving class of compounds with considerable potential for the development of novel anticancer therapies. As demonstrated throughout this review, rational modification of the imidazolium scaffold, including metal coordination, molecular hybridization, and appropriate substitution of the heterocyclic core, enables the design of compounds exhibiting potent antiproliferative activity against a broad spectrum of cancer types through multiple complementary mechanisms of action.

Among the investigated derivatives, metal–NHC complexes, particularly those containing gold(I), platinum(II), and silver(I), consistently demonstrated the highest cytotoxic activity, frequently surpassing the potency of conventional reference drugs. Their biological activity is associated with multitarget mechanisms involving thioredoxin reductase inhibition, oxidative stress induction, mitochondrial dysfunction, DNA damage, immunogenic cell death, and activation of apoptotic signaling pathways. In parallel, hybrid imidazolium derivatives, especially those incorporating lithocholic acid or other biologically active pharmacophores, have emerged as an attractive strategy for simultaneously enhancing cytotoxicity, improving selectivity, and expanding the spectrum of molecular targets. SAR analyses indicate that metal coordination, bulky aromatic or heteroaromatic substituents, molecular hybridization, extended π-conjugation, and balanced lipophilicity represent the most important structural determinants associated with enhanced anticancer activity.

Despite these encouraging advances, the translation of IMSDs into clinical oncology remains at an early stage. Most currently available evidence originates from in vitro studies, whereas comprehensive in vivo investigations, pharmacokinetic characterization, and systematic toxicological evaluation remain limited. Furthermore, interactions with clinically established chemotherapeutic agents, immunotherapy, or radiotherapy have been only marginally investigated, despite the considerable therapeutic potential of combination strategies. These limitations emphasize the need for standardized preclinical evaluation and more comprehensive assessment of safety, efficacy, and mechanisms of action. Future research should therefore extend beyond the synthesis of increasingly potent derivatives and focus on the rational optimization of physicochemical and pharmacological properties, comprehensive safety assessment, validation in clinically relevant animal models, and investigation of combination therapies. In addition, the integration of IMSDs with targeted drug delivery systems, nanotechnology, and multifunctional nanocarriers represents an emerging direction for future development, offering opportunities to improve tumor selectivity, reduce systemic toxicity, and enhance therapeutic efficacy.

Overall, the accumulated evidence suggests that IMSDs represent a potentially valuable platforms in anticancer drug discovery. Continued interdisciplinary efforts integrating medicinal chemistry, chemical biology, pharmacology, materials science, nanotechnology, and translational oncology will be essential to bridge the gap between experimental research and clinical application, ultimately enabling the development of safe, effective, and multifunctional imidazolium-based anticancer therapeutics.

## Figures and Tables

**Table 4:** Cytotoxic activity of NHC and IMSDs against non-cancerous cells.

Experimental Model/Cell Lines		IMSDs	IC_50_/Mechanism/ Results	Selectivity Index (SI)	IC_50_ of Reference Drug	Figure/Ref.
Non-cancer breast cells	MCF-10	1-Benzyl-2-phenyl-3-(4-isopropyl)-benzyl-imidazolium chloride (IBN-65)	Low cytotoxicity IC_50_ = ND	ND	ND	Figure 4A [51]
Lung fibroblasts	IMR90	Imidazolyl Gold(I) Compounds containing triphenylphosphine as a co-ligand	Low cytotoxicity IC_50_ = 63–87 µM	0.88–48.30		Figure 3D [30]
		1-Benzyl-2-phenyl-3-(4-isopropyl)-benzyl-imidazolium chloride (IBN-65)	Low cytotoxicity IC_50_ = ND	ND		Figure 4B [52]
	CCD-19Lu	Platinum (II) complexes with selenophene, Platinum (II) complex with thiophene Platinum (II) complex on a C^N^N scaffold, an NHC ligand derived from benzimidazole	Low cytotoxicity IC_50_ = 3.26–4.94 µM	15.52–164.67	Cisplatin IC_50_ = 38.21μM	Figure 2D [29]
Non- cancer liver cells	THLE-2	1-Benzyl-2-phenyl-3-(4-isopropyl)-benzyl-imidazolium chloride (IBN-65)	Low cytotoxicity IC_50_ = ND	ND	ND	Figure 4A [51]
	LO2	Pt(II)-N-heterocyclic carbene complexes derived from 4,5-diarylimidazole	Promising selectivity IC_50_ = 1.01–12.94 µM	0.05–39.21	Cisplatin IC_50_ = 6.35 μM	Figure 2H [32]
Human dermal fibroblasts	ND	Fe–NHC complexes	Low cytotoxicity IC_50_ = 23.6–42.04 µM	7.24–48.32	ND	Figure 2A [27]
	CRL-1475	Lithocholic acid (LCA)-based imidazolium salts with differing in the number of carbon atoms C1–C16 in the alkyl chain	Low cytotoxicity for the majority of the salts IC_50_ > 100 μg/mL	0.64–5.54		Figure 3D [45,63]
	HaCaT	Au(4,5-dichloro, N-methyl, N′-[2-hydroxy ethylphenyl]imidazol-2-ylide)	At a concentration of 5 μM, the compound was selective, cell viability 70%.	ND	Cisplatin After 24 h the viability > 80%	Figure 3E [33]
Human monocytic cell line	THP1	Lithocholic acid (LCA)-based imidazolium salts with differing in the number of carbon atoms C1–C16 in the alkyl chain	Low cytotoxicity IC_50_ > 77 μg/mL	0.54–14.67	ND	Figure 3D [63]
Mouse fibroblast cells	L-929	Ag (I)-NHC complexes based on 1,3-dioxane ligand	Low cytotoxicity IC_50_ = 11.09–36.38 µM	1.36–11.33	Cisplatin IC_50_ = 36.11 μg/mL	Figure 2E [25]
		Halogen-free Metal-phenolic Imidazolium Poly (Ionic Liquids)	Increased L929 fibroblast proliferation by 22%	ND	ND	Figure 2O [64]
	MEF	2-chloro-1,3-bis(4- nitrobenzyl)-1H-benzo]imidazole-3-ium chloride	Low cytotoxicity IC_50_ = ND	ND	ND	Figure 4C [53]
Non-cancer kidney epithelial cells	Vero-E6	Silver N-heterocyclic biscarbene complexes	Low cytotoxicity IC_50_ = 10.7–26.3 μM	0.38–3.50	Silver sulfadiazine IC_50_ = 15.9 μM	Figure 2G [26]
		Silver N-heterocyclic monocarbene complexes	Low cytotoxicity IC_50_ = 10.3–12.2 μM	0.46–1.23		
Cardiomyocyte cells	H9C2	Lithocholic acid (LCA)-based imidazolium salts with differing in the number of carbon atoms C1–C16 in the alkyl chain	Low cytotoxicity IC_50_ = 52.09–105.4 µg/mL)	0.75–3.99	Doxorubicin IC_50_ = 51.71 µg/mL	Figure 3D [46]

IC_50_ (half maximal inhibitory concentration), ND (not determined).

**Table 5:** Antitumor efficacy and safety of IMSDs in in vivo models.

Type of Cancer (Cell Line)	IC_50_ In Vitro	Animal/N	IMSDs	Administration/Dose	Effect/ Mechanisms	Toxicity	Body Weight Change	Ref.
Ovarian cancer (OVCAR-3)	IC_50_ = 35 µM	Mouse (N = 3)	Ag(I) N-heterocyclic carbene complex obtained from 4,5-dichloro-1H-imidazole,	Subcutaneously at the tumor site every third day for ten days at a dose of 333 mg/kg, for a cumulative dose of 1000 mg/kg over ten days.	Histopathological examination demonstrated necrosis and tumor cell death	Histopathological examination revealed no adverse effects on the brain, liver, lungs, heart, kidneys, or spleen.	ND	[34]
Non-small lung tumor (A549)	IC_50_ = 5 µg/mL	Mouse (N = 8 per group) randomly assigned	1-mesityl-3-(2-naphthoylmethano)-1H-imidazolium bromide (MNIB)	Intraperitoneal injection 10–80 mg/kg	Statistically significant reduction in tumor volume (*p* < 0.05–0.001) compared to the control group	No acute toxicity was detected after administration of MNIB at doses of 10–80 mg/kg.	ND	[65]
Hepatocellular carcinoma (Huh7)	IC_50_ = 5 µM	Balb/c nude mice (N = 8 per group) randomly assigned	1-Benzyl-2-phenyl-3-(4-isopropyl)-benzyl-imidazolium chloride (IBN-65)	Intraperitoneal injection 5 mg/kg once a week for four weeks	Inhibition of angiogenesis (reduction in CD31 expression), reduction in tumor volume of more than 65% H&E staining revealed a reduced amount of fibrous tissue in treated tumors	No apparent systemic toxicity was observed	No statistically significant differences were observed between the treated and control groups	[52]
Hepatocellular carcinoma (Huh7)	IC_50_ = 100 µM	Balb/c nude mice (N = 8 per group) randomly assigned	1,3-bisbenzylimidazolium bromide (IBN-1)	2 g/L in water, mice drink 4 mL water per day	Histopathological analysis showed inhibition of survivin expression and a 31% reduction in tumor size	A 9% reduction in body weight was observed in the treatment group and was considered evidence of systemic toxicity		[51]
Hepatocellular carcinoma (Huh7)	IC_50_ = 100 µM	Balb/c nude mice (N = 8 per group) randomly assigned	1-Mesityl-3-(4-acetate-benzyl)-imidazolium chloride (IBN-9)	0.6 g/L or 1.5 g/L in water, mice drink 4 mL water per day	Histopathological analysis showed inhibition of expression of Ki67, survivin and Cdk4 45% reduction in tumor (dose 0.6 g/L) 60% reduction in tumor (dose 1.5 g/L)	ND	No body weight loss was observed in the treatment groups compared with the control group	[51]
Bladder tumors were induced with 0.05% N-butyl-N-(4-hydroxybutyl)nitrosamine (BBN)	ND	Mouse (N = ND)	Imidazolium salt with a triphenylphosphonium group (TPP1)	Intravesically injection 3000 μM or 1500 μM TPP1	Immunohistochemical analysis revealed increased expression of activated caspase-3 and tumor necrosis	ND	ND	[66]
Colon cancer (HCT116)	IC_50_ = 0.87 µM	Zebrafish (*Danio* *rerio*) (N= 6 per group)	Cationic Fe–NHC complexes	5 µM dissolved in water by 3 days post injection	LD_50_ = 24.624 µM Proliferation about 20%	ND	ND	[27]
	IC_50_ = 3.26 µM		Neutral Fe–NHC complexes	0.25 µM dissolved in water by 3 days post injection	LD50 = 6.861 µM Proliferation about 50%			
Colorectal adenocarcinoma (DLD-1)	Lithocholic acid (LCA)-based imidazolium salt with 6 carbon atom in alkyl chain IC50 DLD-1 = 78.9 μg/mL	Balb/c nude mice (N = 6 per group) randomly assigned	S6- N-Substituent hexyl	Orally administration 100–500 mg/kg	The decrease in TNF-α concentration after administration of 500 mg/kg S6, Increase in Bcl-2 and caspase-3 concentration after administration of 500 mg/kg S6, Histopathological analysis showed 80.2% necrosis and 60% the expression of Ki67 of tumors in groups treated with 500 mg/kg S6	Hematological evaluation of healthy animals showed no toxic effects after administration of 500 mg/kg compared with the control group	No significant changes in body weight were observed compared with baseline	[45]
Breast cancer (MCF-7)	Lithocholic acid (LCA)-based imidazolium salt with 8 carbon atom in alkyl chain S8 IC_50_ (MCF-7 = 13.17 μg/mL)	Balb/c nude mice (N = 10 per group)	S8- N-Substituent octyl	Orally administration 300–500 mg/kg	Histopathological analysis showed reduction in tumor volume compared to control, and doxorubicin. Tumors necrosis > 60%	Animal observation and gross examination of organs at necropsy revealed no signs of toxicity		[46]

N (number of animals per group), IC_50_ (half maximal inhibitory concentration), ND (not determined), LD_50_ (Lethal Dose 50%).

**Table 6:** SAR of IMSDs: influence of structural modifications on anticancer activity and proposed mechanisms of action.

Structural Modification	Observed SAR Trend (Compared to Parent Scaffold)	Effect of Anticancer Activity	Associated Mechanism	Representative Examples (Compounds/Classes)	Refs.
**Aromatic/** **heteroaromatic substituents**	↑ Lipophilicity ↑ π-conjugation ↑ Planarity	↑ Cytotoxicity (lower IC_50_) ↑ Cellular uptake ↑ Selectivity in many cases	DNA binding/intercalation ROS generation Apoptosis induction	Benzyl, naphthyl, acridine, indole, quinoline, benzimidazole derivatives	[41,42,48,50,51,52,54,55,58]
**Alkyl substituents**	↑ Lipophilicity ↑ Membrane permeability Too long →↓ solubility	↑ Activity up to optimal length Too long **↓** selectivity ↑ Nonspecific toxicity	Enhanced uptake Membrane interaction	Methyl, ethyl, propyl, etc., substitutes IMSDs	[43,48,49]
**Hybrid** **pharmacophores**	↑ Structural diversity ↑ Target complementarity ↑ Solubility (in some cases)	↑ Multitarget activity ↑ Selectivity in many studies Moderate to high potency	Apoptosis ROS generation Cell cycle arrest Enzyme inhibition	Curcumin, chalcone, lithocholic acid, coumarin, peptide hybrids	[44,45,46]
**Metal–NHC** **complexes**	↑ Stability of complexes Unique redox properties	Generally high potency (often submicromolar/nanomolar) Broader spectrum (esp. Pt,Ag)	ROS generation/TrxR inhibition DNA damage Mitochondrial dysfunction Apoptosis	Au(I)-NHC, Ag(I)-NHC, Ru(II)-NHC, Pt(II)-NHC, Cu(I/II)-NHC, Fe(II/III)-NHC	[20,21,22,23,24,25,26,27,28,29,30,31,32,33,34,35,37,38,39,40,64]
**Counter anion**	Modulates solubility Affects ion pairing Influences uptake	Activity depending on anion and context Affects selectivity	Indirect: solubility/uptake/membrane transport	PF_6_^−^, BF_4_^−^, Br^−^, Cl^−^, NO_3_^−^	[47,53]

(↑) increase, (↓) decrease, (→) cause.

## Data Availability

No new data were created or analyzed in this study. Data sharing is not applicable to this article.
